# Plasma metabolomics reveals risk factors for lung adenocarcinoma

**DOI:** 10.3389/fonc.2024.1277206

**Published:** 2024-03-19

**Authors:** Mengjie Yu, Wei Wen, Yue Wang, Xia Shan, Xin Yi, Wei Zhu, Jiye Aa, Guangji Wang

**Affiliations:** ^1^ Key Laboratory of Drug Metabolism & Pharmacokinetics, China Pharmaceutical University, Nanjing, Jiangsu, China; ^2^ Department of Thoracic Surgery, First Affiliated Hospital of Nanjing Medical University, Nanjing, Jiangsu, China; ^3^ Department of Respiration, The Affiliated Jiangning Hospital of Nanjing Medical University, Nanjing, Jiangsu, China; ^4^ Department of Oncology, First Affiliated Hospital of Nanjing Medical University, Nanjing, Jiangsu, China

**Keywords:** LUAD, metabolomics, risk factor, oxalate, LDHA

## Abstract

**Background:**

Metabolic reprogramming plays a significant role in the advancement of lung adenocarcinoma (LUAD), yet the precise metabolic changes remain incompletely understood. This study aims to uncover metabolic indicators associated with the progression of LUAD.

**Methods:**

A total of 1083 subjects were recruited, including 670 LUAD, 135 benign lung nodules (BLN) and 278 healthy controls (HC). Gas chromatography-mass spectrometry (GC/MS) was used to identify and quantify plasma metabolites. Odds ratios (ORs) were calculated to determine LUAD risk factors, and machine learning algorithms were utilized to differentiate LUAD from BLN.

**Results:**

High levels of oxalate, glycolate, glycine, glyceric acid, aminomalonic acid, and creatinine were identified as risk factors for LUAD (adjusted ORs>1.2, P<0.03). Remarkably, oxalate emerged as a distinctive metabolic risk factor exhibiting a strong correlation with the progression of LUAD (adjusted OR=5.107, P<0.001; advanced-stage vs. early-stage). The Random Forest (RF) model demonstrated a high degree of efficacy in distinguishing between LUAD and BLN (accuracy = 1.00 and 0.73, F1-score= 1.00 and 0.79, and AUC = 1.00 and 0.76 in the training and validation sets, respectively). TCGA and GTEx gene expression data have shown that lactate dehydrogenase A (LDHA), a crucial enzyme involved in oxalate metabolism, is increasingly expressed in the progression of LUAD. High LDHA expression levels in LUAD patients are also linked to poor prognoses (HR=1.66, 95% CI=1.34-2.07, P<0.001).

**Conclusions:**

This study reveals risk factors associated with LUAD.

## Introduction

Lung cancer is one of the most common and lethal types of malignant tumors worldwide. According to the statistics, there were approximately 2.2 million new cases of lung cancer and 1.8 million deaths worldwide in 2020 (1). Lung adenocarcinoma (LUAD) is the most common histological subtype of lung cancer, accounting for approximately 50% of all lung cancer cases (2). With the development of society, the incidence of various lung nodules, including lung adenocarcinoma, remains high due to environmental pollution, smoking, and unhealthy diet (3, 4). Although the clinical application of low-dose computed tomography (LDCT) has dramatically increased lung nodules’ detection rate and reduced lung adenocarcinoma’s mortality rate, the false-positive rate of lung adenocarcinoma detected by LDCT is high (5). Therefore, there is an unmet need for diagnosing LUAD and accurately classifying lung nodules.

Metabolic reprogramming has been recognized as one of the 10 hallmarks of cancer contributing to tumorigenesis and tumor progression (6–8). Studying the metabolic preferences, physiological dependencies, and molecular mechanisms that underlie LUAD is essential for its diagnosis, progression, and prognosis. For example, a large-scale metabolomic analysis of LUAD showed that combining serum metabolic fingerprints with protein tumor markers by deep learning can be used for early LUAD detection (9). Targeted metabolomic studies of resected lesions deciphered the metabolic trajectory from atypical adenomatous hyperplasia to adenocarcinoma *in situ* and invasive adenocarcinoma, revealing that metabolic perturbations occur in the precancerous lesions of LUAD (10). Extensive research has shown that glucose metabolic pathways, fatty acid metabolism, and glutamine metabolic pathways are associated with the prognosis of LUAD (11–13). Few studies have been performed on how LUAD progresses over time, so characterizing LUAD’s metabolic evolution is necessary from different stages.

This study focused on detecting and screening metabolic markers associated with LUAD. Plasma metabolites from patients with lung nodules (including LUAD and BLN) and their controls were analyzed using a metabolomics platform with GC/MS instrumentation. Metabolic patterns were evaluated, and metabolic markers were screened and described based on semiquantitative data, receiver operating characteristic (ROC) curve analysis and odds ratio (OR). Then, endogenous metabolites and machine learning algorithms were used to construct and evaluate classification models for LUAD. In addition, we found that as the pTNM stages advanced and tumor metastasis, oxalate and its metabolic key enzyme LDHA changed in LUAD. Considering the interaction between oxalate and LDHA, the linkage and functions between the two are briefly explained in the discussion.

## Materials and methods

### Human plasma collection

The experimental samples were collected from the First Affiliated Hospital of Nanjing Medical University from June 2015 to June 2021. Blood samples were collected from 6:00 am to 8:30 am after overnight fasting and kept under 4°C before stored at –80°C within 6 hours after plasma isolation (14). Subjects included in this study were free of metabolic abnormalities such as hypoproteinemia, weight loss, and negative nitrogen balance. This prospective study was approved by the Ethics Committee of the First Affiliated Hospital of Nanjing Medical University (No. 2016-SRFA-149), and informed consent was obtained from all subjects.

### Chemicals and reagents

1, 2-^13^C2-Myristic acid, methyl myristate, methoxamine hydrochloride (purity 98%) and pyridine (≥99.8% GC) were purchased from Sigma-Aldrich (St. Louis, MO, USA). N-methyl-trimethylsilyltrifluoroacetamide (MSTFA) and 1% trimethylchlorosilane (TMCS) were provided by Pierce Chemical (Rockford, IL, USA). Methanol and *n*-heptane were HPLC grade and obtained from Merck (Darmstadt, Germany).

### GC/MS analysis, instrumental setting, and parameters

Plasma samples were processed, extracted, and derived in accordance with our previously developed methods (15). 50.0 μL plasma was added into 200.0 μL methanol (containing 1, 2-^13^C_2_-Myristic acid, 5.0 µg/mL). The specimens were vigorously extracted for 5.0 min and centrifuged at 20000×g for 10.0 min at 4°C. A 100.0 μL aliquot of the resulting supernatant was transferred to a GC vial and evaporated to dryness in a Speed-Vac concentrator (Thermo Fisher Scientific, Savant™ SC250EXP, Holbrook, USA). The dried plasma samples were then methoxymated, where 30.0 μL of methoxyamine pyridine solution (10.0 mg/mL) was added to the residue and incubated for 16 h at room temperature. Then the samples were trimethylsilylated for another 1.0 h by adding 30.0 μL of MSTFA with 1% TMCS as a catalyst. At last, 30.0 μL n-heptane with methyl myristate (15.0 µg/mL) was added to each GC vial as an external standard to monitor the stability of GC/MS.

A 0.5 μL sample aliquot was injected into gas chromatography coupled to a mass spectrometer (Shimadzu GCMS-QP2010 Ultra, Kyoto, Japan) in split mode (split ratio 8:1). It was equipped with an Rtx-5MS capillary column (0.25 mm × 30 m × 0.25µm, Restek, PA, USA). The injector temperature was set at 250°C. Helium was used as the carrier gas at a 1.5 mL/min flow rate. The column temperature was initially kept at 80°C for 3.0 min, then raised to 300°C at a rate of 20°C/min, and held for 5.0 min. The mass spectrometer ion source temperature and interface temperature were both 220°C, and ions were generated by a 70-eV electron beam at a current of 3.2 mA. The mass spectra were acquired over the mass range of 50-700 m/z in a full scan mode, with each run of 19.0 min. Following the abovementioned procedure, the quality control (QC) samples were prepared for the plasma pool. All samples were randomly selected for GC-MS analysis to diminish the systematic variations.

The metabolites were by comparing the mass spectrum and retention indexes for the analyte with the corresponding values from the literature and various libraries [e.g., Mainlib and Public in the National Institute of Standards and Technology (NIST) library 2.0 (2008) and Wiley 9 (Wiley-VCH Verlag GmbH & Co KGaA, Weinheim, Germany)]. Some standard compounds were also utilized to identify the metabolites.

### Statistical analysis

After normalization against the internal standard, all the semiquantitative data from GC/MS were log_10_-transformed. The transformed data were imported into SIMCA-P 14.1 software (Germany, Sartorius, Goettingen) and pre-processed for multivariate statistical analysis using par scaling. Principal component analysis (PCA) and partial least square to latent structure discriminant analysis (PLS-DA) models were built and plotted to show the clustering or separation of samples from different groups. PLS-DA models were constructed and plotted to show the clustering or separation of samples from different groups. The goodness of fit for the PLS-DA models was evaluated using three quantitative parameters: R^2^X, R^2^Y and Q^2^. R^2^X and R^2^Y are the explained variations, and Q^2^ is the predicted variation, with a higher level of R^2^Y and Q^2^Y indicating the model’s better fit and predictive performance. To avoid the classification obtained by supervised learning methods being chance and to test whether the model reproduces well and whether the data in the model are over-fitted, the validity of the built model was examined by a 7-fold cross-check and replacement test (200 times, cross-validation). The intercept of the R^2^ and Q^2^ regression lines to the axes was used to measure overfitting, and the model was valid when the intercept of Q^2^ was negative.

To assess the differences among the groups, analysis of variance (ANOVA) and multiple comparisons (LSD) were used. The independent-sample t-test and Mann-Whitney U test were used to analyze normally and non-normally distributed data. OR calculations and ROC curve analysis were performed using SPSS 26.0 (SPSS Inc., Chicago, IL, USA), bar graphs were produced using GraphPad Prism 8.0, and heatmap and pathway analysis were performed using the online software MetaboAnalyst (https://www.metaboanalyst.ca/).

### Model development and evaluation

All individuals were randomly divided into train and validation datasets in a ratio of seven to three. Hyperparameters of seven machine learning models [including XGBoost, AdaBoost, Random Forest (RF), Gaussian Naive Bayes (GNB), Multilayer Perceptron (MLP), Support Vector Machine (SVM), and k-Nearest Neighbors (KNN)] were optimized using 10-fold cross-validation. Ten groups were created randomly from the training set. In each iteration of the 10-fold cross-validation method, nine groups were randomly selected for training and the remaining groups were used as test sets. Thus, the test sets for each group were selected sequentially, which ensured that the evaluation results did not overlap. Then, to minimize errors due to unreasonable test set selection, the results of the 10 evaluations were averaged.

The ROC curve analysis, calibration curve, decision curve analysis (DCA), accuracy, F1-score, sensitivity and specificity were used to assess the model’s performance. Model discrimination was assessed with ROC analysis, and the accuracy of its predictions was assessed with AUC. The calibration curve showed the calibration and the extent to which the model’s predictions deviated from actual events. Clinical utility and net benefit were assessed with DCA, which allows estimating the net benefit by calculating the difference between the true positive rate and the false positive rate, weighted by the odds ratio of the selected risk threshold probabilities.

All analyses were performed with R software (version 4.0) and Python version 3.7.

### Transcriptomics database

RNA-sequencing expression (level 3) profiles and corresponding clinical information for lung adenocarcinoma were downloaded from the TCGA dataset (https://portal.gdc.com). The current-release (V8) GTEx datasets were obtained from the GTEx data portal website (https://www.gtexportal.org/home/datasets). Statistical analyses were performed using R software v4.0.3 (R Foundation for Statistical Computing, Vienna, Austria). P-value <0.05 was considered statistically significant. All analyses were performed using the online website HOME for Researchers (https://www.home-for-researchers.com/static/index.html#/).

### Cell culture

A549 and BEAS-2B cell lines were purchased from the Type Culture Center, Chinese Academy of Sciences (Shanghai, China). These cell lines were grown in RPMI-1640 medium supplemented with 10% (v/v) fetal bovine serum and 100 U/mL penicillin and streptomycin at 37°C and 5% CO2. GC/MS and metabolomics analysis methods were the same as previously reported (16).

## Results

### Metabolic phenotypes of LUAD and the controls

**Figure 1:**
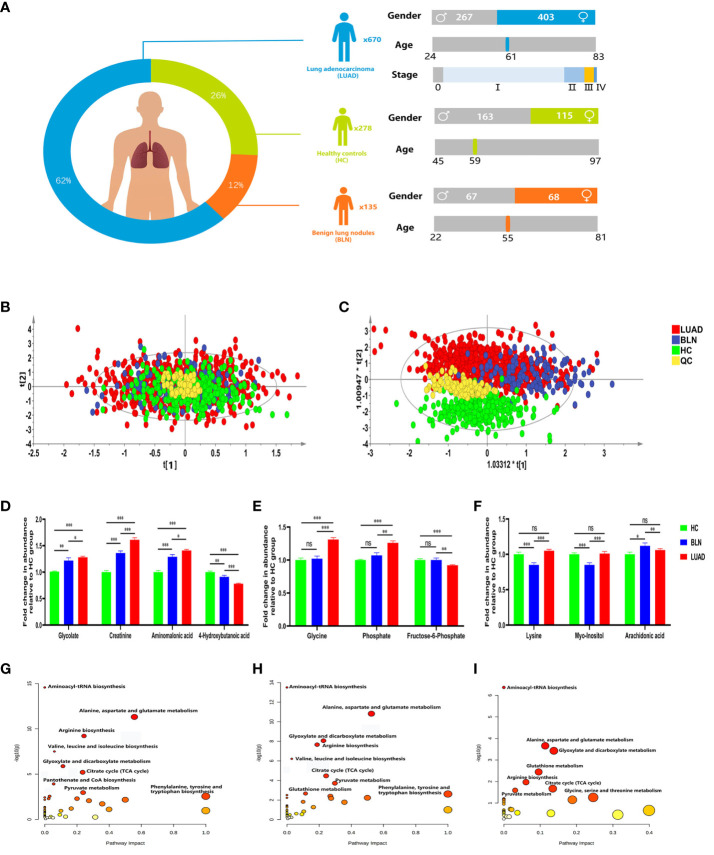
Distribution of subjects in this study and analysis of metabolic differences. **(A)** Distribution of subjects included in this study. **(B)** PCA score plot. **(C)** PLS-DA score plot. **(D)** Relative abundance of plasma glycolate, creatinine, aminomalonic acid and 4-hydroxybutanoic acid. **(E)** Relative abundance of plasma glycine, phosphate and fructose-6-phosphate. **(F)** Relative abundance of plasma lysine, myo-inositol and arachidonic acid (*, **and *** to denote 0.01 ≤ p < 0.05, 0.001 ≤ p < 0.01, and p < 0.001, respectively). **(G)** Metabolic pathway analysis of identified differential metabolites in BLN and HC groups; **(H)** Metabolic pathway analysis of identified differential metabolites in LUAD and HC groups; **(I)** Metabolic pathway analysis of identified differential metabolites in LUAD and BLN groups.

A total of 1083 subjects were included in this study, including 670 lung adenocarcinoma (LUAD), 135 benign lung nodules (BLN) and 278 healthy controls (HC). Benign lung nodules mainly include pulmonary hamartomas, hemangiomas, and inflammatory pseudotumors. LUAD and BLN participants were newly diagnosed and had not undergone anti-cancer treatment, including radiation therapy, chemotherapy, surgical intervention, or medication administration. All patients included in this study were diagnosed by pathological examination. The distribution of subjects is shown in **Figure 1A** and **Supplementary Table 1**.

GC/MS analysis of the plasma samples aligned the metabolites in typical chromatograms (**Supplementary Figure 1**). Deconvolution of the GC/MS chromatograms produced 103 independent peaks from the plasma samples, 54 of which were authentically identified as metabolites (**Supplementary Table 2**). Quantitative data were acquired for each metabolite in the plasma samples of the HC, BLN and LUAD cases.

The PCA and PLS-DA score plots (**Figures 1B, C**) demonstrated good clustering of pooled QC samples, indicating reproducibility of the assay and consistent instrument performance throughout the experiment. The supervised PLS-DA model showed that the samples in the HC and lung nodule groups (including LUAD and BLN) were distributed in different quadrants (**Figure 1C**), indicating significant metabolic differences between the two groups. Similarly, the overlap between LUAD and BLN indicates similar metabolic models. R^2^X, R^2^Y and Q^2^ of the PLS-DA model were 0.628, 0.552 and 0.527, respectively, which suggested that the PLS-DA model had good adaptability and predictability. The permutation plot (**Supplementary Figure 2**) demonstrated that the PLS-DA models were valid: the Q^2^ regression line had a negative intercept, and all of the permuted R^2^ values to the left of the intercept were lower than the original point to the right. These results suggest that both variability and similarity in metabolic patterns are present in the three groups.

### Metabolic features of LUAD

Based on statistical analysis (**Table 1**), 43 and 42 discriminant metabolites were found to differentiate LUAD and BLN patients from healthy controls, respectively. Similarly, LUAD cases primarily showed different metabolomic patterns from BLN patients. According to the statistical analysis, 26 distinct metabolites were identified between LUAD and BLN.

**Table 1:** List of discriminant metabolites: BLN vs. HC, LUAD vs. HC and LUAD vs. BLN.

Metabolites	HC (n=278)	BLN (n=135)	LUAD (n=670)	P1	P2	P3
Pyruvate	4.66 ± 0.16	4.58 ± 0.37	4.65 ± 0.42	<0.001	0.038	ns
Lactate	6.39 ± 0.21	6.32 ± 0.23	6.37 ± 0.36	<0.001	0.002	<0.001
Glycolate	3.86 ± 0.10	3.91 ± 0.17	3.93 ± 0.15	0.009	<0.001	0.020
Alanine	6.34 ± 0.20	6.26 ± 0.18	6.32 ± 0.17	<0.001	<0.001	0.004
Oxalate	5.38 ± 0.15	5.53 ± 0.17	5.42 ± 0.21	<0.001	<0.001	<0.001
3-Hydroxybutyric acid	4.82 ± 0.32	4.65 ± 0.33	4.67 ± 0.30	<0.001	<0.001	ns
Monomethylphosphate	4.53 ± 0.13	4.53 ± 0.15	4.54 ± 0.16	ns	0.017	ns
Valine	5.32 ± 0.15	5.25 ± 0.13	5.27 ± 0.14	<0.001	<0.001	ns
4-Hydroxybutanoic acid	4.84 ± 0.19	4.78 ± 0.18	4.71 ± 0.20	0.001	<0.001	<0.001
Phosphate	6.09 ± 0.06	6.09 ± 0.14	6.14 ± 0.18	ns	<0.001	0.004
Leucine	5.91 ± 0.15	5.86 ± 0.13	5.87 ± 0.13	<0.001	<0.001	ns
Isoleucine	5.51 ± 0.27	5.47 ± 0.21	5.47 ± 0.24	<0.001	<0.001	ns
Proline	5.63 ± 0.34	5.52 ± 0.33	5.59 ± 0.37	<0.001	0.032	0.012
Glycine	4.90 ± 0.20	4.92 ± 0.18	5.00 ± 0.22	ns	<0.001	<0.001
Succinate	3.80 ± 0.13	3.65 ± 0.18	3.64 ± 0.24	<0.001	<0.001	ns
Glyceric acid	3.92 ± 0.08	3.97 ± 0.17	4.00 ± 0.17	0.005	<0.001	ns
Fumarate	4.12 ± 0.17	4.07 ± 0.16	4.07 ± 0.24	0.007	<0.001	ns
Serine	5.52 ± 0.18	5.43 ± 0.14	5.47 ± 0.14	<0.001	<0.001	0.004
Threonine	5.15 ± 0.17	5.09 ± 0.12	5.11 ± 0.13	<0.001	<0.001	0.025
β-Alanine	4.97 ± 0.03	4.98 ± 0.03	4.97 ± 0.02	0.021	ns	0.017
Aminomalonic acid	5.42 ± 0.21	5.55 ± 0.17	5.59 ± 0.16	<0.001	<0.001	0.018
Malate	3.99 ± 0.10	3.80 ± 0.23	3.85 ± 0.25	<0.001	<0.001	0.018
Aspartate	4.36 ± 0.15	4.25 ± 0.22	4.29 ± 0.24	<0.001	<0.001	ns
Methionine	4.72 ± 0.14	4.65 ± 0.13	4.64 ± 0.14	<0.001	<0.001	ns
Pyroglutamate	5.63 ± 0.07	5.53 ± 0.24	5.61 ± 0.30	<0.001	<0.001	0.012
Cysteine	4.44 ± 0.10	4.42 ± 0.12	4.43 ± 0.12	0.036	ns	ns
Creatinine	4.45 ± 0.21	4.60 ± 0.17	4.67 ± 0.17	<0.001	<0.001	<0.001
Glutamate	5.29 ± 0.20	5.24 ± 0.23	5.26 ± 0.29	0.014	0.009	ns
Phenylalanine	5.22 ± 0.13	5.17 ± 0.11	5.17 ± 0.12	<0.001	<0.001	ns
Pyrophosphoric acid	5.10 ± 0.11	4.99 ± 0.13	5.03 ± 0.18	<0.001	<0.001	<0.001
Asparagine	4.41 ± 0.17	4.27 ± 0.15	4.32 ± 0.17	<0.001	<0.001	0.002
Glutamine	6.05 ± 0.13	5.91 ± 0.12	5.94 ± 0.13	<0.001	<0.001	0.003
Citrate	5.69 ± 0.10	5.58 ± 0.10	5.61 ± 0.10	<0.001	<0.001	0.008
Ornithine	5.00 ± 0.22	4.83 ± 0.25	4.95 ± 0.30	<0.001	0.006	<0.001
Glucose	5.89 ± 0.25	5.93 ± 0.55	5.84 ± 0.52	ns	0.040	ns
Lysine	4.53 ± 0.20	4.46 ± 0.18	4.54 ± 0.20	<0.001	ns	<0.001
Tyrosine	5.58 ± 0.13	5.52 ± 0.13	5.52 ± 0.12	<0.001	<0.001	ns
Palmitelaidic acid	4.08 ± 0.27	4.03 ± 0.31	4.04 ± 0.30	0.045	0.008	ns
Palmitic acid	5.71 ± 0.10	5.63 ± 0.12	5.66 ± 0.13	<0.001	<0.001	0.027
Uric acid	5.80 ± 0.15	5.73 ± 0.20	5.72 ± 0.18	<0.001	<0.001	ns
Myo-Inositol	4.74 ± 0.12	4.67 ± 0.12	4.73 ± 0.15	<0.001	ns	<0.001
Linoleic acid	5.15 ± 0.13	5.01 ± 0.17	5.04 ± 0.18	<0.001	<0.001	ns
Oleic acid	5.57 ± 0.15	5.57 ± 0.14	5.56 ± 0.15	ns	0.046	ns
Cystine	3.93 ± 0.23	4.00 ± 0.30	3.96 ± 0.27	0.028	ns	ns
Fructose-6-Phosphate	3.88 ± 0.14	3.88 ± 0.14	3.84 ± 0.15	ns	<0.001	0.005
Arachidonic acid	4.23 ± 0.18	4.27 ± 0.19	4.23 ± 0.21	0.016	ns	0.003
Monopalmitin	4.32 ± 0.11	4.16 ± 0.11	4.21 ± 0.12	<0.001	<0.001	<0.001
Alpha-Tocopherol	5.12 ± 0.12	4.90 ± 0.26	4.89 ± 0.35	<0.001	<0.001	ns
Cholesterol	5.86 ± 0.06	5.84 ± 0.08	5.83 ± 0.14	0.013	0.003	ns

The data were log10 transformed and expressed as mean ± SD. P1, BLN vs. HC; P2, LUAD vs. HC; P3, LUAD vs. BLN; ns, P>0.05.

Of the 42 metabolites differentiating BLN from HC, the levels of glycolate, creatinine and aminomalonic acid were higher in BLN, while 4-hydroxybutanoic acid was lower, and all the above metabolites deviated further in LUAD (**Figure 1D**). These findings indicate that the above metabolites are involved in the development of lung nodules (from averagely minimal damage to lung cancer). Although glycine, phosphate and fructose-6-phosphate showed significant differences between the LUAD and BLN groups, they had no significant difference between the BLN and HC groups (**Figure 1E**). It is, therefore, suggested that these markers are associated with LUAD. In addition, BLN cases showed deviations in lysine, myo-inositol, and arachidonic acid levels, whereas the LUAD and HC groups did not show any significant differences (**Figure 1F**), suggesting that they are markers associated with BLN.

Metabolic pathway analysis showed that patients with lung nodules were more affected by the following metabolic pathways: aminoacyl-tRNA biosynthesis, alanine, aspartate and glutamate metabolism, glyoxylate and dicarboxylate metabolism, citrate cycle, arginine biosynthesis (**Figures 1G, H**). The differential metabolic pathways between LUAD patients and BLN were: aminoacyl-tRNA biosynthesis, alanine, aspartate and glutamate metabolism, glyoxylate and dicarboxylate metabolism, glutathione metabolism, arginine biosynthesis (**Figure 1I**).

### Potential risk factors for LUAD

The ROC analysis showed monopalmitin, succinate, glutamine, alpha-tocopherol, malate, asparagine and pyroglutamate performed well for HC and BLN differentiation (AUC ≥ 0.80) (**Supplementary Table 3**). Meanwhile, succinate and creatinine performed well for the differentiation of HC and LUAD (AUC ≥ 0.80) (**Supplementary Table 4**). However, each metabolite performed poorly in differentiating LUAD and BLN (AUC < 0.65) (**Supplementary Table 5**).

ORs values were calculated to assess the role of the above metabolites as risk factors for predicting BLN/LUAD occurrence (**Table 2**). Glycolate, oxalate, glyceric acid, β-alanine, aminomalonic acid, creatinine, cystine and arachidonic acid were found to be risk factors for BLN (ORs>1.0, P<0.05). In the meantime, glycolate, oxalate, glycine, glyceric acid, aminomalonic acid and creatinine were risk factors for LUAD (ORs>1.3, P<0.05). These results suggest that high levels of glycolate, oxalate, glyceric acid, aminomalonic acid and creatinine were independent risk factors of lung nodules. Whereas glycine may be a LUAD-specific risk factor, similarly, β-alanine, cystine and arachidonic acid may be BLN-specific risk factors.

**Table 2:** List of risk factors: BLN vs. HC, LUAD vs. HC and LUAD vs. BLN.

Cohorts	Risk factors	OR	95%CI	P-value	OR (adjusted)	95%CI (adjusted)	P-value (adjusted)
**BLN vs. HC**	Glycolate	2.283	[1.386, 3.761]	0.001	2.43	[1.464, 4.034]	0.001
	Oxalate	10.37	[5.911, 18.193]	<0.001	11.437	[6.356, 20.579]	<0.001
	Glyceric acid	3.081	[1.762, 5.390]	<0.001	3.139	[1.786, 5.518]	<0.001
	β-Alanine	13.991	[1.307, 149.738]	0.029	12.874	[1.171, 141.589]	0.037
	Aminomalonic acid	2.855	[1.991, 4.092]	<0.001	2.952	[2.033, 4.284]	<0.001
	Creatinine	3.659	[2.465, 5.430]	<0.001	3.861	[2.570, 5.802]	<0.001
	Cystine	1.045	[0.998, 1.095]	0.061	1.042	[0.994, 1.093]	0.09
	Arachidonic acid	1.533	[1.081, 2.174]	0.017	1.547	[1.083, 2.210]	0.016
**LUAD vs. HC**	Creatinine	2.892	[2.089, 4.003]	<0.001	3.408	[2.413, 4.813]	<0.001
	Oxalate	1.394	[1.107, 1.756]	0.005	1.287	[1.031, 1.607]	0.026
	Glycine	1.985	[1.610, 2.448]	<0.001	2.055	[1.656, 2.551]	<0.001
	Glyceric acid	3.211	[2.302, 4.479]	<0.001	3.204	[2.281, 4.502]	<0.001
	Aminomalonic acid	4.62	[3.511, 6.079]	<0.001	4.371	[3.307, 5.778]	<0.001
	Creatinine	6.319	[4.768, 8.373]	<0.001	6.473	[4.850, 8.639]	<0.001
**LUAD vs. BLN**	Alanine	1.771	[1.267, 2.475]	0.001	1.71	[1.222, 2.393]	0.002
	Phosphate	1.824	[1.222, 2.722]	0.003	1.935	[1.283, 2.919]	0.002
	Proline	1.158	[1.007, 1.331]	0.04	1.174	[1.018, 1.353]	0.027
	Glycine	1.768	[1.346, 2.322]	<0.001	1.812	[1.371, 2.395]	<0.001
	Serine	2.052	[1.339, 3.146]	0.001	2.337	[1.510, 3.616]	<0.001
	Threonine	1.597	[1.031, 2.474]	0.036	1.858	[1.182, 2.922]	0.007
	Aminomalonic acid	1.535	[1.094, 2.152]	0.013	1.47	[1.040, 2.080]	0.029
	Malate	1.316	[1.034, 1.676]	0.026	1.296	[1.019, 1.647]	0.034
	Pyroglutamate	1.356	[1.077, 1.708]	0.01	1.406	[1.113, 1.777]	0.004
	Creatinine	1.956	[1.412, 2.710]	<0.001	1.993	[1.425, 2.787]	<0.001
	Pyrophosphoric acid	1.35	[1.006, 1.811]	0.045	1.474	[1.091, 1.993]	0.012
	Asparagine	1.773	[1.240, 2.537]	0.002	2.033	[1.401, 2.951]	<0.001
	Glutamine	1.935	[1.246, 3.007]	0.003	2.193	[1.393, 3.454]	0.001
	Ornithine	1.596	[1.299, 1.960]	<0.001	1.64	[1.326, 2.028]	<0.001
	Lysine	1.857	[1.382, 2.494]	<0.001	1.982	[1.463, 2.686]	<0.001
	Palmitic acid	1.752	[1.119, 2.742]	0.014	1.69	[1.069, 2.672]	0.025
	Myo-Inositol	2.871	[1.802, 4.573]	<0.001	2.429	[1.495, 3.945]	<0.001
	Monopalmitin	2.803	[1.781, 4.411]	<0.001	2.954	[1.860, 4.693]	<0.001

All ORs were adjusted for age and gender.

Analyzing the differential metabolites between LUAD and BLN (**Table 2**), we found that alanine, phosphate, proline, glycine, serine, threonine, aminomalonic acid, malate, pyroglutamate, creatinine, pyrophosphoric acid, asparagine, glutamine, ornithine, lysine, palmitic acid, myo-inositol, monopalmitin 18 substances may be risk factors for the development of BLN to LUAD (ORs>1.1, P<0.05).

### Oxalate characterizes the progression of LUAD

The occurrence and progression of lung adenocarcinoma are progressive processes over time. Identifying the metabolic changes during lung adenocarcinoma progression is highly important for diagnosing, treating, and prognosis. The pTNM staging is the most commonly used method of tumor staging, which mainly consists of three parts: T-primary tumor size, N-lymph node metastasis and M-distant metastasis. We set to stage 0, stage I and stage II as early-stage, stage III and stage IV as advanced-stage, considering the progression of LUAD and pTNM staging.

**Figure 2:**
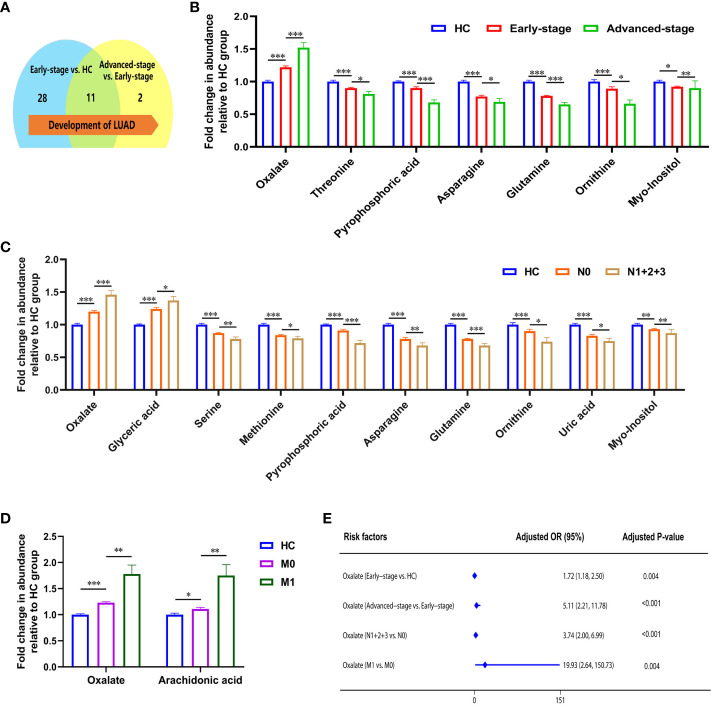
Metabolic features associated with the progression of lung adenocarcinoma. **(A)** Changes in the number of differential metabolites during lung adenocarcinoma development. **(B)** Relative abundance of asparagine, myo-inositol, ornithine, pyrophosphoric acid, threonine, and glutamine decreased, and oxalate increased in HC, early-stage, and advanced-stage patients. **(C)** Relative abundance of oxalate, glyceric acid, nonanoic acid, and arachidonic acid were elevated in patients with lymphatic metastasis, and levels of phosphate, proline, glycine, serine, methionine, creatinine, pyrophosphoric acid, asparagine, glutamine, ornithine, lysine, uric acid, and myo-inositol were decreased (N1 + 2+3, patients with lymphatic metastasis; N0, patients without lymphatic metastasis). **(D)** Relative abundance of oxalate and arachidonic acid in patients with distant metastatic (M1, patients with distant metastatic; M0, patients without distant metastatic). *, **and *** to denote 0.01 ≤ p < 0.05, 0.001 ≤ p < 0.01, and p < 0.001, respectively. **(E)** Odds ratio values of oxalate.

Statistical analysis showed that the metabolism in lung adenocarcinoma changes with tumorigenesis and progression (**Table 3**). Thirty-nine differential metabolites were found between early-stage LUAD and HC groups, and thirteen between advanced-stage LUAD and early-stage LUAD (**Figure 2A**). Notably, plasma asparagine, myo-inositol, ornithine, pyrophosphoric acid, threonine, and glutamine levels gradually decreased with increasing pTNM staging, while oxalate was elevated (**Figure 2B**). OR values suggested that oxalate may be a risk factor for progressive exacerbation of LUAD disease (**Supplementary Table 6**). These findings suggest that the above metabolites may be implicated in the development and progression of LUAD (from early to the advanced stage).

**Table 3:** List of discriminant metabolites: early-stage vs. HC and advanced-stage vs. early-stage.

Metabolites	HC (n=278)	Early-stage (n=475)	Advanced-stage (n=39)	P1	P2
Glycolate	3.86 ± 0.10	3.92 ± 0.15	3.84 ± 0.17	<0.001	0.007
Oxalate	5.38 ± 0.15	5.45 ± 0.21	5.56 ± 0.14	<0.001	<0.001
Glycine	4.90 ± 0.20	4.97 ± 0.20	4.85 ± 0.21	<0.001	<0.001
Fumarate	4.12 ± 0.17	4.04 ± 0.21	4.10 ± 0.18	<0.001	0.017
Threonine	5.15 ± 0.17	5.11 ± 0.13	5.07 ± 0.11	<0.001	0.042
Aminomalonic acid	5.42 ± 0.21	5.58 ± 0.16	5.51 ± 0.16	<0.001	0.006
Pyrophosphoric acid	5.10 ± 0.11	5.03 ± 0.20	4.92 ± 0.17	<0.001	<0.001
Asparagine	4.41 ± 0.17	4.30 ± 0.16	4.25 ± 0.17	<0.001	0.026
Glutamine	6.05 ± 0.13	5.94 ± 0.13	5.86 ± 0.13	<0.001	<0.001
Ornithine	5.00 ± 0.22	4.92 ± 0.28	4.80 ± 0.26	<0.001	0.014
Myo-Inositol	4.74 ± 0.12	4.71 ± 0.12	4.66 ± 0.18	0.011	0.005
β-Alanine	4.97 ± 0.03	4.97 ± 0.02	4.96 ± 0.02	ns	0.003
Arachidonic acid	4.23 ± 0.18	4.24 ± 0.22	4.36 ± 0.24	ns	0.004
Pyruvate	4.66 ± 0.16	4.60 ± 0.40	4.67 ± 0.37	<0.001	ns
Lactate	6.39 ± 0.21	6.33 ± 0.39	6.35 ± 0.21	<0.001	ns
Alanine	6.34 ± 0.20	6.30 ± 0.15	6.28 ± 0.15	<0.001	ns
3-Hydroxybutyric acid	4.82 ± 0.32	4.67 ± 0.31	4.71 ± 0.32	<0.001	ns
Valine	5.32 ± 0.15	5.25 ± 0.15	5.23 ± 0.10	<0.001	ns
4-Hydroxybutanoic acid	4.84 ± 0.19	4.71 ± 0.21	4.76 ± 0.27	<0.001	ns
Leucine	5.91 ± 0.15	5.86 ± 0.12	5.84 ± 0.11	<0.001	ns
Isoleucine	5.51 ± 0.27	5.47 ± 0.21	5.32 ± 0.47	<0.001	ns
Proline	5.63 ± 0.34	5.58 ± 0.35	5.42 ± 0.53	0.005	ns
Succinate	3.80 ± 0.13	3.61 ± 0.21	3.64 ± 0.28	<0.001	ns
Glyceric acid	3.92 ± 0.08	4.00 ± 0.16	4.05 ± 0.15	<0.001	ns
Serine	5.52 ± 0.18	5.46 ± 0.13	5.43 ± 0.11	<0.001	ns
Malate	3.99 ± 0.10	3.82 ± 0.22	3.88 ± 0.28	<0.001	ns
Aspartate	4.36 ± 0.15	4.27 ± 0.23	4.24 ± 0.23	<0.001	ns
Methionine	4.72 ± 0.14	4.65 ± 0.12	4.63 ± 0.12	<0.001	ns
Pyroglutamate	5.63 ± 0.07	5.56 ± 0.24	5.59 ± 0.23	<0.001	ns
Creatinine	4.45 ± 0.21	4.65 ± 0.16	4.58 ± 0.20	<0.001	ns
Glutamate	5.29 ± 0.20	5.22 ± 0.28	5.27 ± 0.22	<0.001	ns
Phenylalanine	5.22 ± 0.13	5.16 ± 0.11	5.18 ± 0.11	<0.001	ns
Citrate	5.69 ± 0.10	5.62 ± 0.10	5.60 ± 0.11	<0.001	ns
Tyrosine	5.58 ± 0.13	5.51 ± 0.11	5.52 ± 0.11	<0.001	ns
Palmitelaidic acid	4.08 ± 0.27	4.02 ± 0.30	3.96 ± 0.35	<0.001	ns
Palmitic acid	5.71 ± 0.10	5.65 ± 0.13	5.66 ± 0.12	<0.001	ns
Uric acid	5.80 ± 0.15	5.71 ± 0.18	5.67 ± 0.22	<0.001	ns
Linoleic acid	5.15 ± 0.13	5.04 ± 0.18	5.07 ± 0.17	<0.001	ns
Fructose 6-phosphate	3.88 ± 0.14	3.85 ± 0.14	3.86 ± 0.12	0.009	ns
Monopalmitin	4.32 ± 0.11	4.19 ± 0.11	4.21 ± 0.11	<0.001	ns
Alpha-Tocopherol	5.12 ± 0.12	4.89 ± 0.37	4.87 ± 0.35	<0.001	ns

The data were log10 transformed and expressed as mean ± SD. P1, Early-stage vs. HC; P2, Advanced-stage vs. Early-stage; ns, P>0.05.

The correlation between metabolites and tumor size was analyzed with Spearman. Ten metabolites (alanine, valine, phosphate, leucine, asparagine, glutamine, ornithine, lysine, palmitic acid, and linoleic acid) were negatively correlated with tumor size (**Supplementary Figure 3**). With the appearance of lymphatic metastases, plasma levels of oxalate, glyceric acid, nonanoic acid, and arachidonic acid increased in LUAD, and phosphate, proline, glycine, serine, methionine, creatinine, pyrophosphoric acid, asparagine, glutamine, ornithine, lysine, uric acid, and myo-inositol were decreased (**Figure 2C**; **Supplementary Table 7**). In addition, high plasma levels of oxalate and glyceric acid may be risk factors for lymphatic metastasis in lung adenocarcinoma (**Supplementary Table 7**). Significant differences were observed in the glycolate, oxalate, glycine, and arachidonic acid levels between LUAD with distant metastasis and non-distant metastases (**Supplementary Table 8**). High levels of oxalate and arachidonic acid may be prognostic biomarkers for distant metastasis in lung adenocarcinoma (**Figure 2D**; **Supplementary Table 8**).

In summarizing the changes in metabolites during lung adenocarcinoma progression, it is clear that patients’ metabolic profiles are changing. As the pTNM stages advanced and tumor metastasis, plasma oxalate levels increased, indicating that oxalate is closely associated with lung adenocarcinoma progression. Notably, OR values suggested that plasma oxalate may be a risk marker of the progression in LUAD (**Figure 2E**).

### Development and evaluation of machine learning predictive models

**Figure 3:**
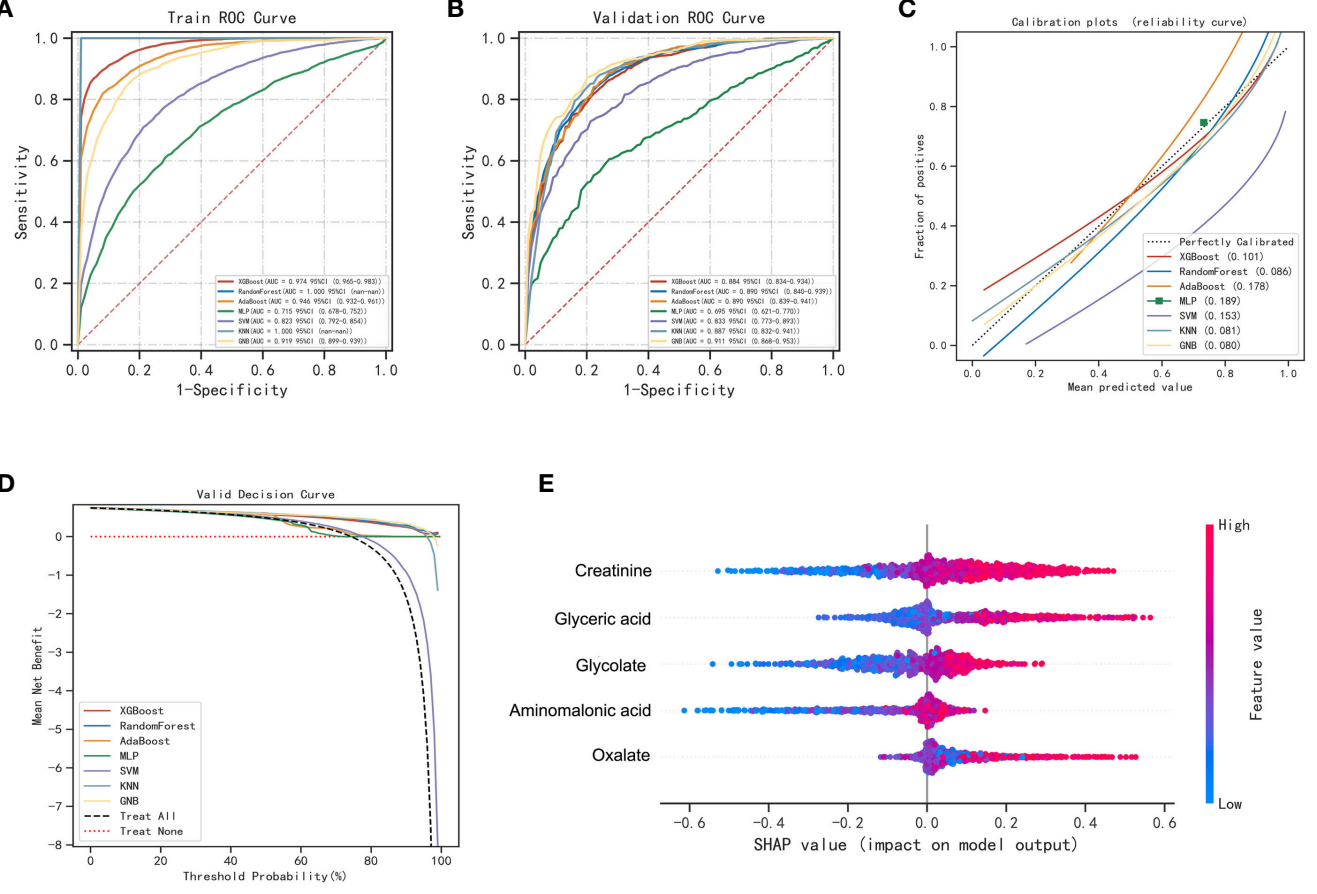
Performance of various machine learning models in pulmonary nodules and healthy controls. **(A)** The ROC curves of the training set. **(B)** The ROC curves of the validation set. **(C)** The calibration plots of the validation set. Each graph’s 45° straight line perfectly matches the observed (y-axis) and predicted (x-axis) survival probabilities. A closer distance between two curves indicates greater accuracy. **(D)** Decision curve analysis graph showing the net benefit against threshold probabilities based on decisions from model outputs. **(E)** Shapley additive explanation (SHAP) summary plot of 5 feature clusters, derived by aggregating related values of a particular feature (e.g., the average, minimum, and maximum). Each dot corresponds to the SHAP value of the feature cluster for the lung cancer risk score of a given case patient or control subject at a certain point in time. A feature’s SHAP value (x-axis) represents the contribution of the specific feature to the risk score, with positive values indicating a contribution that increases the risk score and negative values indicating a contribution that lowers the score. The location of the dot on the x-axis represents its SHAP value, whereas its color represents the cluster’s value (the actual value of the feature that is represented in the cluster), with red representing higher values (for features measured along a continuum) or affirmative responses (for binary features). The dots are piled up vertically to show their density. The feature clusters are sorted by their mean absolute SHAP values.

To improve the differential diagnostic performance between HC, BLN and LUAD, we developed AI-based prediction models using seven machine learning algorithms (including XGBoost, RF, AdaBoost, MLP, SVM, KNN and GNB). Glycolate, oxalate, glyceric acid, aminomalonic acid and creatinine were used to build predictive models for lung nodules and healthy controls. The results are shown in **Figure 3**; **Supplementary Table 9**. Compared with others, the RF model performs best in both the training and validation set (accuracy =1.00 and 0.85; F1-score =1.00 and 0.87; AUC=1.00 and 0.89, respectively). The calibration curves showed high consistency between the validation cohorts’ predicted and observed survival probability. The DCA results showed that the RF model had an excellent net benefit across the whole range of threshold probabilities. Meanwhile, the RF models also showed excellent differentiation effects for distinguishing BLN or LUAD from HC, respectively (**Supplementary Figures 4**, **5**). These results suggested that the RF model has the best diagnostic accuracy and applicability for distinguishing lung nodules from healthy controls.

**Figure 4:**
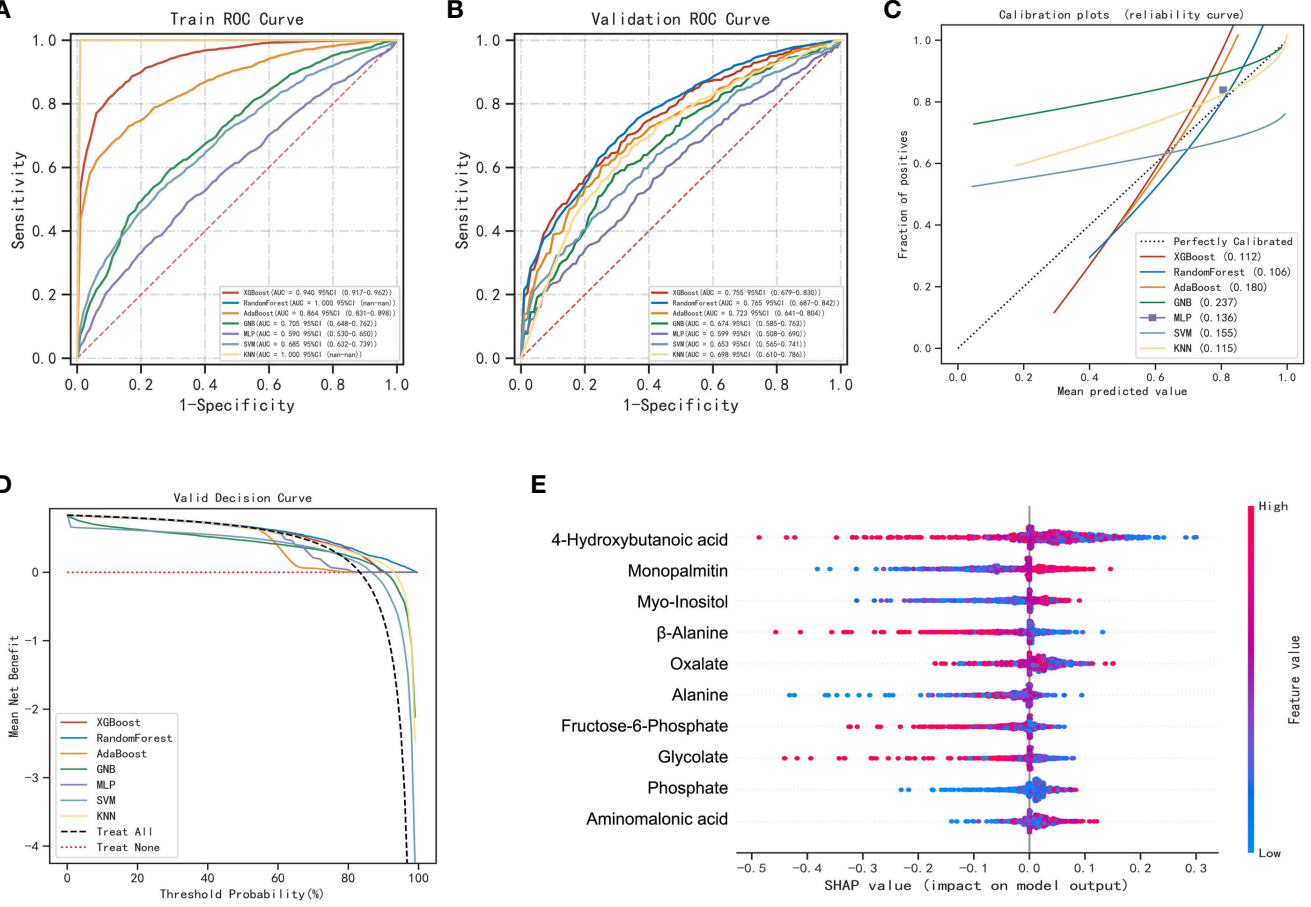
Performance of various machine learning models in LUAD and BLN groups. **(A)** The ROC curves of the training set. **(B)** The ROC curves of the validation set. **(C)** The calibration plots of the validation set. **(D)** Decision curve analysis graph showing the net benefit against threshold probabilities based on decisions from model outputs. **(E)** SHAP summary plot of 10 feature clusters.

4-hydroxybutanoic acid, monopalmitin, myo-inositol, β-alanine, oxalate, alanine, fructose-6-phosphate, glycolate, phosphate, and aminomalonic acid were screened out to construct prediction models for LUAD and BLN (**Supplementary Figure 6**). The results are shown in **Figure 4**; **Supplementary Table 10**. Compared with others, the RF model performs best in both the training and validation set (accuracy =1.00 and 0.73; F1-score =1.00 and 0.79; AUC=1.00 and 0.76, respectively). The calibration curves showed high consistency between the validation cohorts’ predicted and observed survival probability. The DCA results showed that the RF model had an excellent net benefit across the whole range of threshold probabilities. These results suggested that the RF model can distinguish LUAD from BLN with high performance and accuracy.

### RF modeling and plasma oxalate in an external validation set

**Figure 5:**
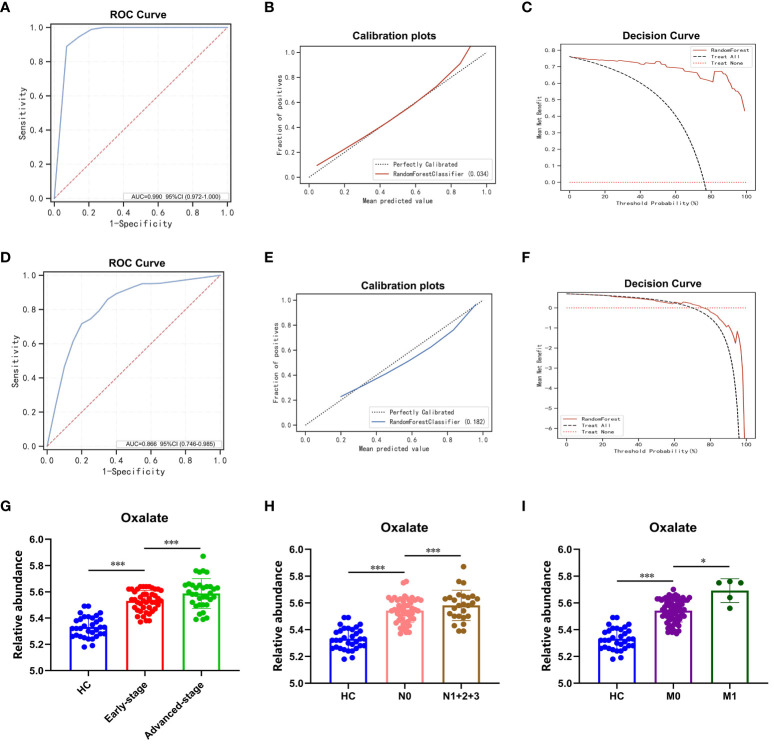
RF modeling and plasma oxalate in the external validation set. **(A)** The ROC curve of the external validation set comparing pulmonary nodules and healthy controls. **(B)** The calibration plots of the external validation set comparing pulmonary nodules and healthy controls. **(C)** The DCA plots of the external validation set comparing pulmonary nodules and healthy controls. **(D)** The ROC curve of the external validation set comparing LUAD and BLN. **(E)** The calibration plots of the external validation set comparing LUAD and BLN. **(F)** The DCA plots of the external validation set comparing LUAD and BLN. **(G)** Plasma oxalate increased in HC, early-stage, and advanced-stage LUAD. **(H)** Plasma oxalate was elevated in LUAD with lymphatic metastasis (N1 + 2+3, patients with lymphatic metastasis; N0, patients without lymphatic metastasis). **(I)** Plasma oxalate in LUAD with distant metastatic (M1, patients with distant metastatic; M0, patients without distant metastatic). * and *** to denote 0.01 ≤ p < 0.05 and p < 0.001, respectively.

A sample set was obtained from the Affiliated Hospital of Nanjing University Medical School in Nanjing, China for the purpose of external validation. This sample set comprised of 33 healthy controls, 23 patients with benign lung nodules, and 77 patients diagnosed with LUAD. The RF model previously developed was assessed on this distinct sample set, with the outcomes presented in **Figure 5**. The model exhibited high accuracy in predicting healthy controls and benign lung nodules, achieving an AUC of 0.99 (**Figure 5A**). The calibration curve and decision curve analysis further validated the model’s precision and substantial net benefit (**Figures 5B, C**). In patients with benign lung nodules and lung adenocarcinoma, the model demonstrated an AUC of 0.866, indicating its relevance to these specific subgroups (**Figures 5D, F**). A noteworthy finding of the study was the correlation between elevated plasma oxalate levels and advanced pTNM stage and tumor metastasis in LUAD patients, as evidenced by an independent external validation set, underscoring the significant association between oxalate and LUAD progression (**Figures 5G–I**).

### Alterations in pivotal enzymes involved in the metabolic pathway of oxalate

The primary sources of oxalate in humans are exogenous dietary intake (20-50%) and endogenous synthesis (50-80%) (17). The subjects collected in this study were mainly from Jiangsu Province, China, and there was slight dietary variation between subjects. Although ascorbic acid has been shown to have an essential effect on oxalate production, the process proceeds mainly through a non-enzymatic reaction, and the mechanism of action is unknown (18). In addition, we have ruled out the possibility of additional ascorbic acid supplementation in LUAD patients during treatment.

**Figure 6:**
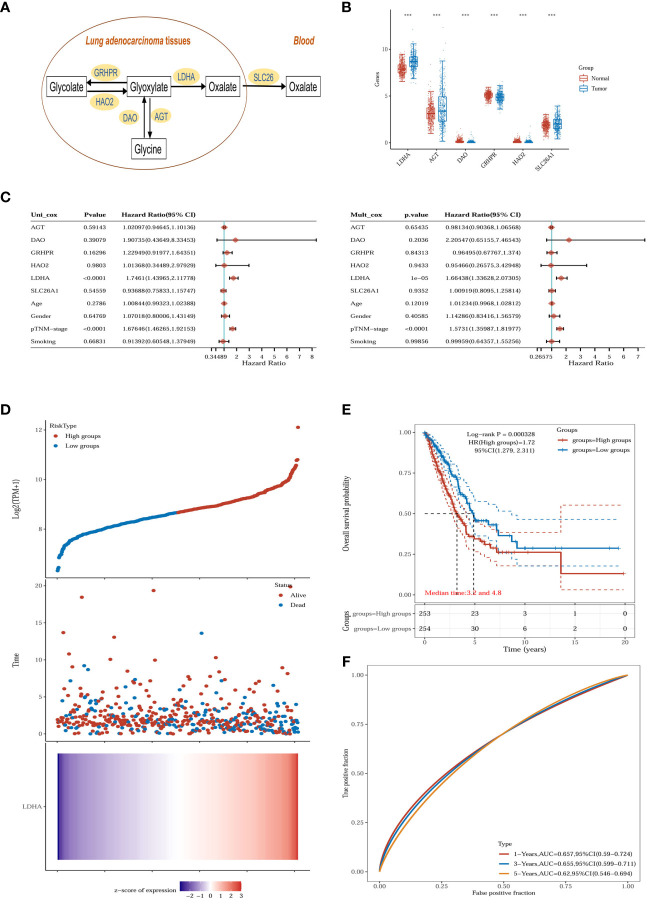
Oxalate metabolism in lung adenocarcinoma. **(A)** Oxalate-related metabolism in LUAD. **(B)** The oxalate-related enzymes [lactate dehydrogenase A (LDHA), glyoxylate reductase/hydroxypyruvate reductase (GRHPR), alanine–glyoxylate aminotransferase (AGT), d-amino acid oxidase (DAO), hydroxy acid oxidase 2 (HAO2) and solute carrier family 26 member 1 (SLC26A1)] are significantly changed in the tumor (n = 516) compared with normal tissue (n = 637). ***p < 0.001. **(C)** The p-value, risk coefficient (HR) and confidence interval are analyzed by univariate and multivariate Cox regression. **(D)** The gene expression, survival time and survival status of the TCGA dataset. The top scatterplot represents the gene expression from low to high. Different colors represent different groups. The scatter plot distribution represents the gene expression of different samples correspond to the survival time and survival status. The bottom Figure is the gene expression heatmap. **(E)** Kaplan-Meier survival analysis of the gene signature from the TCGA dataset, comparison among different groups was made by log-rank test. HR (High exp) represents the hazard ratio of the low-expression sample relatives to the high-expression sample. HR> 1 indicates the gene is a risk factor, and HR<1 indicates the gene is a protective factor.HR(95%Cl), the median survival time (LT50) for different groups. **(F)** The ROC curve of the gene. The higher values of AUC correspond to higher predictive power.

Glyoxalate is the primary precursor of endogenous oxalate synthesis (19). TCGA and GTEx database results showed significant differences in the expression of enzymes and transporters related to oxalate metabolism (LDHA, GRHPR, AGT, DAO, HAO2 and SLC26A1) in tumor tissues of lung adenocarcinoma patients (**Figures 6A, B**). Univariate and multivariate Cox regression analyses showed (**Figure 6C**) that LDHA, a key enzyme in the biosynthesis of oxalate, was significantly associated with the overall survival (OS) of LUAD patients and was a separately available prognostic indicator (HR = 1.66, 95% CI = 1.34 - 2.07, P < 0.001). A risk score was then calculated for each patient based on LDHA expression levels and risk factors, and patients were classified into low-risk and high-risk (**Figure 6D**). The heatmap revealed that high-risk patients tended to express LDHA genes at high levels, and low-risk patients tended to express LDHA genes at low levels (**Figure 6D**). Survival curves showed that patients with low-risk scores significantly had longer survival times than those with high-risk scores (**Figure 6E**). ROC analysis showed that LDHA could predict prognosis (**Figure 6F**).

**Figure 7:**
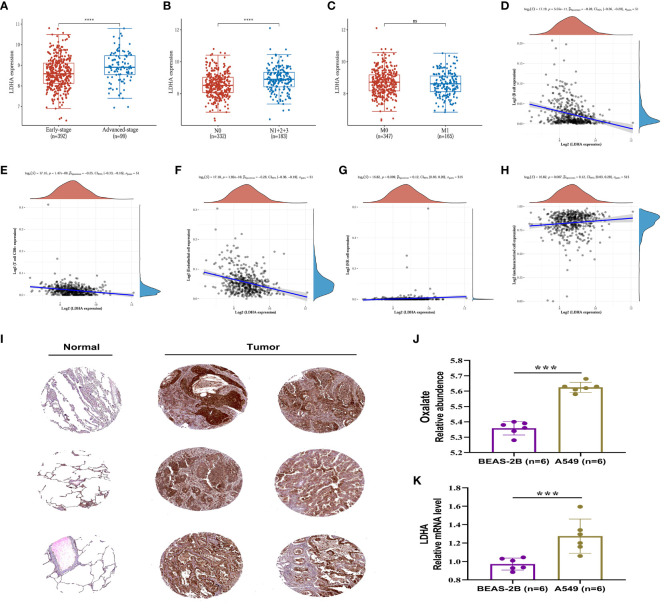
Expression and immune correlation analysis of LDHA in lung adenocarcinoma. **(A)** Expression distribution of LDHA gene in early-stage and advanced-stage LUAD tumor tissues. **(B)** Expression distribution of LDHA gene in LUAD tumor tissues of lymphatic metastasis and non-lymphoid metastasis groups. **(C)** Expression distribution of LDHA gene in LUAD tumor tissues of distant and non-distant metastasis groups. ****p < 0.0001, asterisks (*) stand for significance levels. **(D–H)** The correlations between LDHA gene expression and the immune score were analyzed with Spearman. The abscissa represents the distribution of the LDHA gene expression, and the ordinate represents the distribution of the immune score. The density curve on the right represents the trend in the distribution of the immune score, and the upper-density curve represents the trend in the distribution of the gene expression or the score. The value on the top represents the correlation p-value, correlation coefficient and correlation calculation method. **(I)** (IHC) The Immunohistochemistry (IHC) labelling images of normal lung tissue and tumor tissue were obtained from The Human Protein Atlas (http://www.proteinatlas.org/). **(J)** The relative content of oxalate in BESA-2B and A549 cells. **(K)** Expression of LDHA in BESA-2B and A549 cells. ***p < 0.001; ‘ns’ stands for non-significant, indicating that P > 0.05.

The expression of LDHA in tumor tissues of lung adenocarcinoma increases with the advancement of the pTNM stage and lymphatic metastasis, suggesting that LDHA may be associated with abnormal immune function (**Figures 7A–C**). The European prospective investigation into cancer and nutrition (EPIC) array was used to analyze the correlation between immune cells and LDHA in the TCGA dataset. LDHA was negatively correlated with the expression of B cells, T cell CD8+, and endothelial cells (**Figures 7D–F**) and positively correlated with the expression of NK cells and uncharacterized cells (**Figures 7G, H**). In conclusion, LDHA is positively associated with the progression and prognosis of lung adenocarcinoma. The performance of LDHA further demonstrates the strong potential of oxalate as a risk factor of tumor progression in lung adenocarcinoma.

In order to assess the LDHA expression levels in LUAD, the protein expression of the LDHA gene was analyzed through immunohistochemistry, utilizing data from the Human Protein Atlas (HPA) database (https://www.proteinatlas.org/). The results indicated a significantly higher expression of LDHA in lung adenocarcinoma tissue compared to normal lung tissue (**Figure 7I**).

### Oxalate and LDHA in lung adenocarcinoma cells

The relative content of oxalate and the expression of LDHA were further examined and analyzed in A549 and BEAS-2B cells. The results, depicted in **Figures 7J, K**, indicated a significantly higher oxalate content in A549 cells compared to BEAS-2B cells, accompanied by elevated LDHA expression. These findings suggest that increased LDHA and oxalate levels may represent a specific alteration in LUAD.

## Discussion

Researchers have reported that genetic and microenvironmental factors drive clonal evolution within tumors, leading to metabolic liabilities and facilitating cancer progression (20, 21). Identifying the metabolic profile of lung adenocarcinoma is essential to understanding its progression and diagnosis. This study screened risk factors for LUAD by metabolomic analysis of plasma samples. We applied the machine learning algorithm to metabolite data to develop prediction models for lung nodules and LUAD. We confirmed that plasma oxalate is a risk factor associated with LUAD progression and further explored the interaction between oxalate and LDHA.

We analyzed plasma metabolites and confirmed the metabolic differences and similarities between lung adenocarcinoma and benign lung nodules. For metabolic differences, glycine and arachidonic acid may be LUAD and BLN-specific risk factors. The abnormal glycine metabolism has been widely reported in cancer (22, 23), so elevated plasma glycine in LUAD may be a common manifestation of cancer. Significantly, elevated plasma glycine may be an important phenomenon that distinguishes LUAD from benign nodules/healthy individuals. Surprisingly, arachidonic acid did not change significantly in the early-stage LUAD but was significantly elevated in BLN and advanced-stage LUAD (**Tables 1**, **3**; **Supplementary Tables 9**, **10**). Thus, plasma arachidonic acid may be important in distinguishing early-stage lung adenocarcinoma from benign lung nodules. This variable profile may be related to the degree of inflammatory response and abnormal immune response function. Based on the differences in metabolic patterns, we identified two panels of metabolites to distinguish LUAD from BLN/HC. Surprisingly, the machine learning models constructed with these two panels showed good prediction accuracy and diagnostic performance.

For metabolic similarity, we found that glycolate, oxalate, glyceric acid, aminomalonic acid and creatinine were elevated in both LUAD and BLN, showing potential as risk factors for lung nodules (with LUAD and BLN). Furthermore, a panel composed of these five metabolites showed excellent diagnostic performance for differentiating lung nodules from healthy controls. Interestingly, except for oxalate, the levels of the other four substances did not consistently increase with the exacerbation of LUAD. These results suggested that changes in glycolate, glyceric acid, aminomalonic acid, and creatinine may be co-occurring changes associated with the development of lung nodules. Machine learning models built from these metabolites could be used for the early screening of lung nodules.

With the improvement of technology and the availability of various kinds of big data, combining omics with machine learning algorithms has attracted increasing attention from the scientific community (24). Recently, machine learning algorithms (such as deep learning, random forest, and XGBoost) were applied to lung cancer research because of their advantages, which include high precision, robustness, simple operation, and fast response (9, 25–27). These machine learning methods have their merits and demerits, so choosing the best methods that best suit our research is crucial. Seven commonly used machine learning algorithms were applied to this study. We found that the random forest model performed best by evaluating various metrics such as prediction accuracy, AUCs, positive predictive values, and negative predictive values. Machine learning models are considered ‘black boxes,’ which can be considered a limitation of the study. In this study, calibration curves, DCA analysis curves, and SHAP values were constructed to maximize model credibility and transparency. Generally, we were very rigorous in model screening, evaluation, and validation. Finally, we constructed an early screening model for lung nodules and a diagnostic model for lung adenocarcinoma using machine learning algorithms.

The present study is the first to identify oxalate as a risk factor closely associated with lung adenocarcinoma progression. Combining plasma metabolites and gene expression results from the TCGA and GTEx databases, and we found that elevated oxalate may be associated with LDHA overexpression. Many studies have documented elevated oxalate in tumor tissues and blood of lung cancer, and inhibition of LDHA expression can effectively reduce oxalate production (28–31). Therefore, it can be concluded that the overexpression of LDHA may cause an increase in plasma oxalate in lung adenocarcinoma patients, and inhibition of LDHA may reduce oxalate production.

LDHA is a promising therapeutic target for various malignancies, which plays important roles in tumorigenesis, progression, invasion and metastasis (32). Surprisingly, although LDHA promotes oxalate production, oxalate is a competitive inhibitor of LDHA (33). Several studies have shown that oxalate has anticancer effects on various cancer cell lines, including liver, breast, colorectal, lymphoma, medulloblastoma and ovarian cancer (33–38). In addition, oxalate has been shown to inhibit cell proliferation and migration and promote oxidative phosphorylation and epithelial-to-mesenchymal transition (35, 39). However, the oxalate concentrations required for significant therapeutic effects are too high for clinical use (40, 41). Because oxalate is an ionized conjugate base that readily complexes with divalent cations such as Mg^2+^ and Ca^2+^ (42). Hence, it could be hypothesized that free oxalate may have a potential protective effect and oxalate elevation may be a negative feedback-like ‘protective response’ against LDHA overexpression. However, high oxalate concentrations can lead to the formation of oxalate crystals, limiting its inhibitory effect on LDHA. Therefore, increasing the concentration of free oxalate and reducing the formation of oxalate crystals may be a new idea to inhibit LDHA expression and improve the prognosis of LUAD.

## Limitations

This study analyzed the metabolic profile of patients with lung adenocarcinoma, but some limitations remain. The inclusion of solely Jiangsu Province, China subjects has resulted in a relatively small sample size, and it is possible that these results will not be reproducible on a wide scale across regions and populations. The mechanism of action between oxalate and LDHA was superficially investigated in this study, and further studies are needed to follow.

## Conclusion

We analyzed the plasma metabolic profile of lung adenocarcinoma and provided an understanding of the progress of LUAD. This study has important implications for further translating basic research into more accurate diagnosis and treatment for clinical purposes.

## Data availability statement

Publicly available datasets were analyzed in this study. This data can be found here: dbGAP database ID at TCGA (phs000178.v11.p8) and GTEx (phs000424.v9.p2).

## Ethics statement

The studies involving humans were approved by the Ethics Committee of the First Affiliated Hospital of Nanjing Medical University. The studies were conducted in accordance with the local legislation and institutional requirements. The participants provided their written informed consent to participate in this study. Written informed consent was obtained from the individual(s) for the publication of any potentially identifiable images or data included in this article. This prospective study was approved by the Ethics Committee of the First Affiliated Hospital of Nanjing Medical University (No. 2016-SRFA-149), and informed consent was obtained from all subjects.

## Author contributions

MY: Writing – original draft, Data curation. WW: Writing – review & editing, Validation, Project administration. YW: Writing – review & editing, Methodology, Data curation. XS: Writing – review & editing, Investigation, Conceptualization. XY: Writing – review & editing, Software. WZ: Writing – review & editing, Resources, Project administration. JA: Writing – review & editing, Visualization, Resources, Funding acquisition. GW: Writing – review & editing, Visualization, Resources, Funding acquisition.
