# Supplementary material for: Plasma metabolomics reveals risk factors for lung adenocarcinoma
Source: Front Oncol. 2024 Mar 19;14:1277206. doi: 10.3389/fonc.2024.1277206 (PMC10985191; doi:10.3389/fonc.2024.1277206)
Supplement: Supplementary file 1 [file DataSheet_1.pdf]

Plasma Metabolomics Reveals Risk Factors for Lung Adenocarcinoma

Mengjie Yu[1,#], Wei Wen[2,#], Yue Wang[1,#], Xia Shan[3], Xin Yi[1], Wei Zhu[4,*], Jiye Aa [1,*] & Guangji Wang[1]

[1]Key Laboratory of Drug Metabolism & Pharmacokinetics, China Pharmaceutical University, Nanjing, Jiangsu Province 210009, China

[2]Department of thoracic surgery, First Affiliated Hospital of Nanjing Medical University, Guangzhou Road, Nanjing, Jiangsu Province 210029, China

[3]Department of Respiration, the Affiliated Jiangning Hospital of Nanjing Medical University, Nanjing, Jiangsu 210000, China

[4]Department of Oncology, First Affiliated Hospital of Nanjing Medical University, Guangzhou Road, Nanjing, Jiangsu 210029, China

*Correspondence: Jiye Aa, Key Laboratory of Drug Metabolism & Pharmacokinetics, China Pharmaceutical University, Nanjing, Jiangsu Province 210009, China. Email: jiyea@cpu.edu.cn. Wei Zhu, Department of Oncology, First Affiliated Hospital of Nanjing Medical University, Guangzhou Road, Nanjing, Jiangsu 210029, China. Email: zhuwei@njmu.edu.cn.

[#] Mengjie Yu, Wei Wen and Yue Wang contributed equally to this work.

Supplementary figures

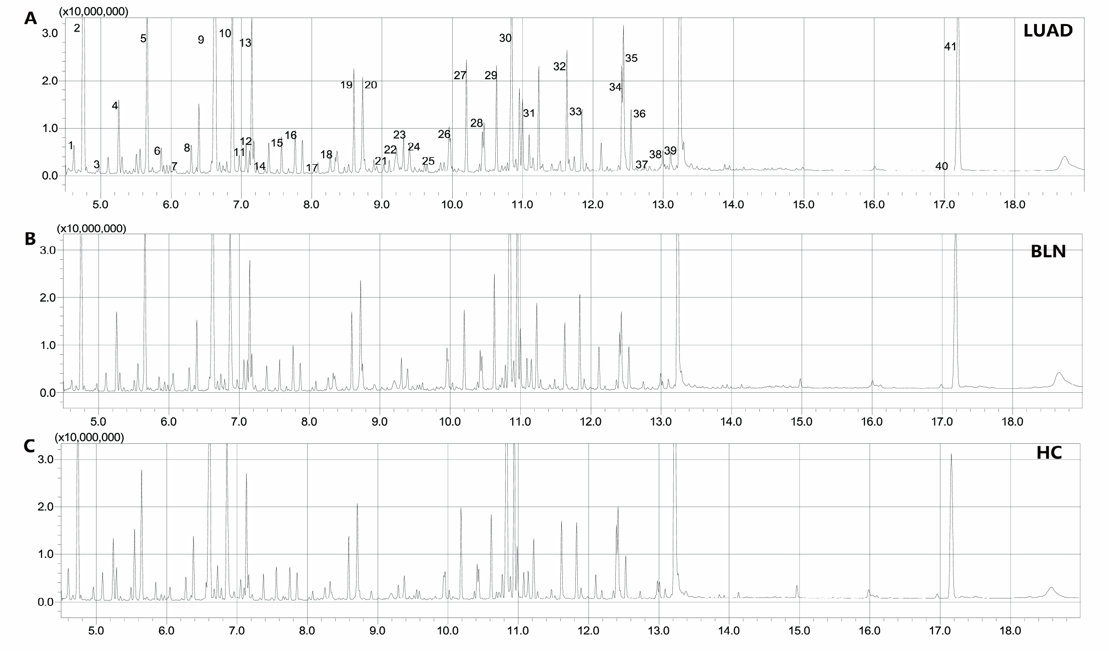

Supplementary Figure 1. Typical GC/MS chromatograms and identified molecules in the plasma from the LUAD (A), the BLN (B) and the HC (C). 1, Pyruvate; 2, Lactate; 3, Glycolate; 4, Alanine; 5, Oxalate; 6, 3-Hydroxybutyric acid; 7, Monomethylphosphate; 8, Valine; 9, Urea; 10, Phosphate; 11, Isoleucine; 12, Proline; 13, Glycine; 14, Succinate; 15, Serine; 16, Threonine; 17, β-Alanine; 18, Aminomalonic acid; 19, Aspartate; 20, Methionine; 21, Cysteine; 22, Ornithine; 23, Glutamate; 24, Phenylalanine; 25, Asparagine; 26, ES; 27, Glutamine; 28, Citrate; 29, IS; 30, Glucose; 31, Tyrosine; 32, Palmitic acid; 33, Uric acid; 34, Linoleic acid; 35, Oleic acid; 36, Octadecanoic acid; 37, Cystine; 38, Fructose-6-Phosphate; 39, Arachidonic acid; 40, Alpha-Tocopherol; 41, Cholesterol.

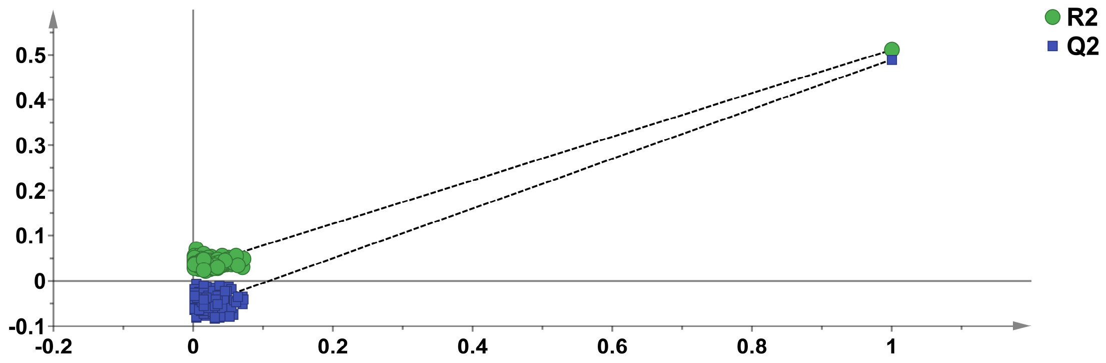

Supplementary Figure 2. Permutation test score plots.

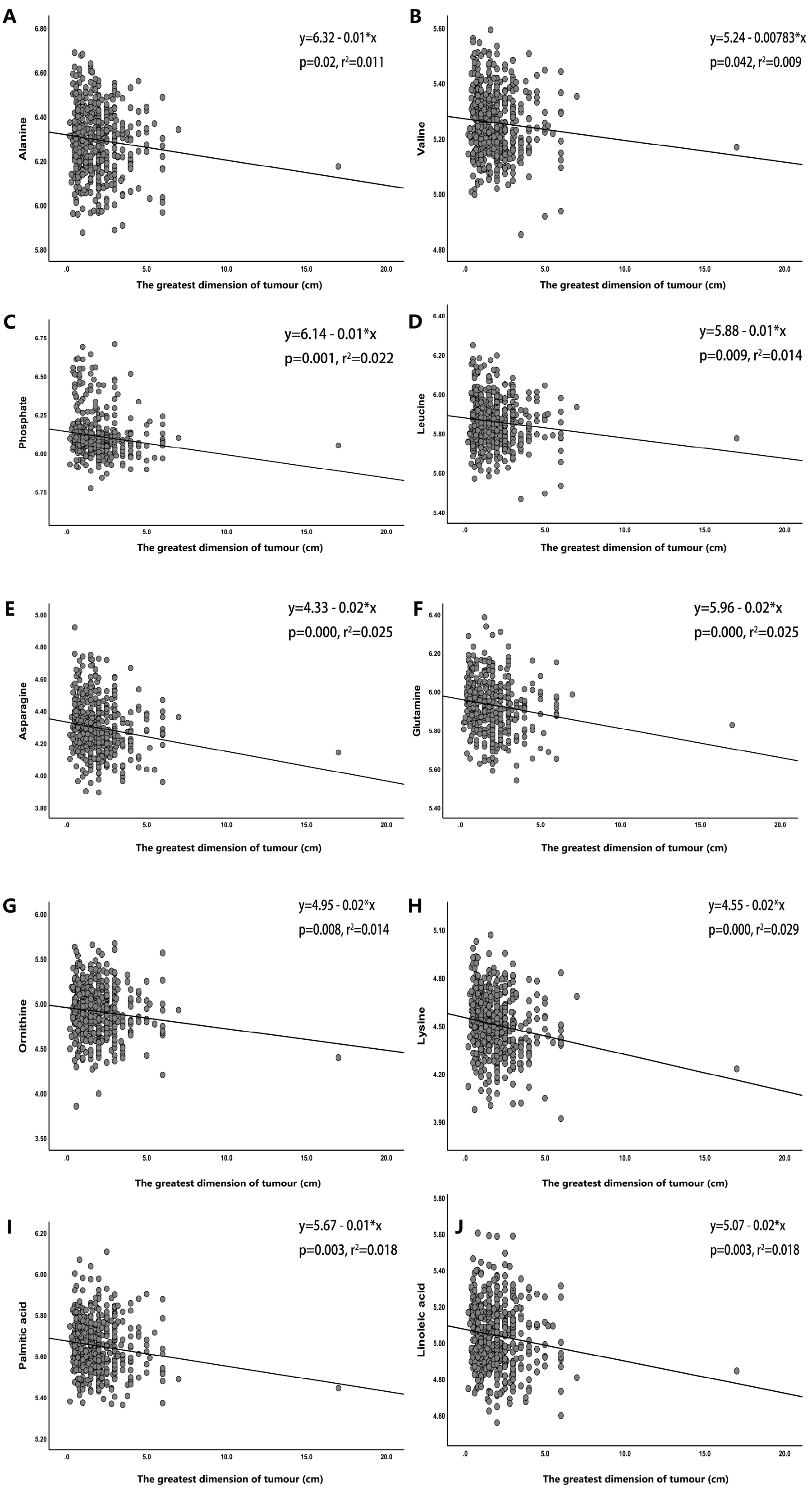

Supplementary Figure 3. Correlation between lung adenocarcinoma tumor size and plasma metabolites.

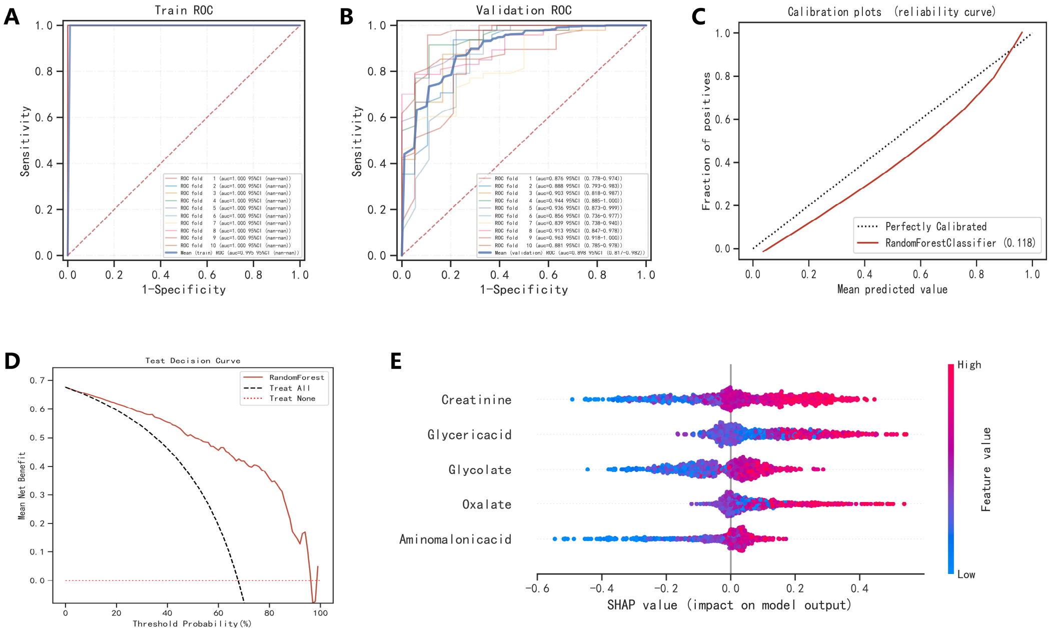

Supplementary Figure 4. Performance of the random forest model in LUAD and HC groups. (A) The ROC curves of the training set. (B) The ROC curves of the validation set. (C) The calibration plots of the validation set. (D) Decision curve analysis graph showing the net benefit against threshold probabilities based on decisions from model outputs. (E) SHAP summary plot of 5 feature clusters.

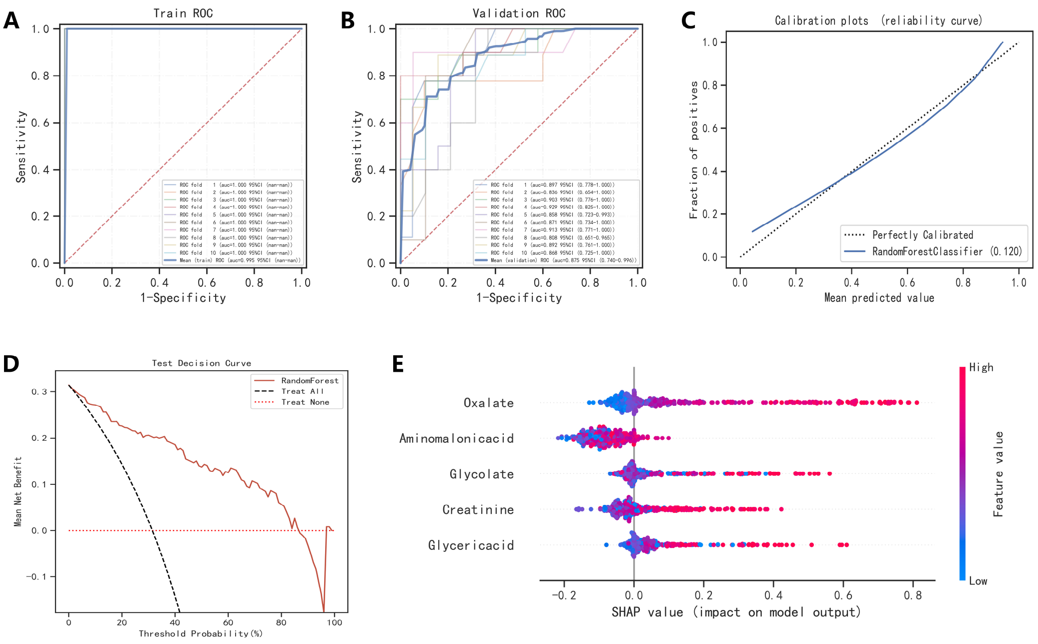

Supplementary Figure 5. Performance of the random forest model in BLN and HC groups. (A) The ROC curves of the training set. (B) The ROC curves of the validation set. (C) The calibration plots of the validation set. (D) Decision curve analysis graph showing the net benefit against threshold probabilities based on decisions from model outputs. (E) SHAP summary plot of 5 feature clusters.

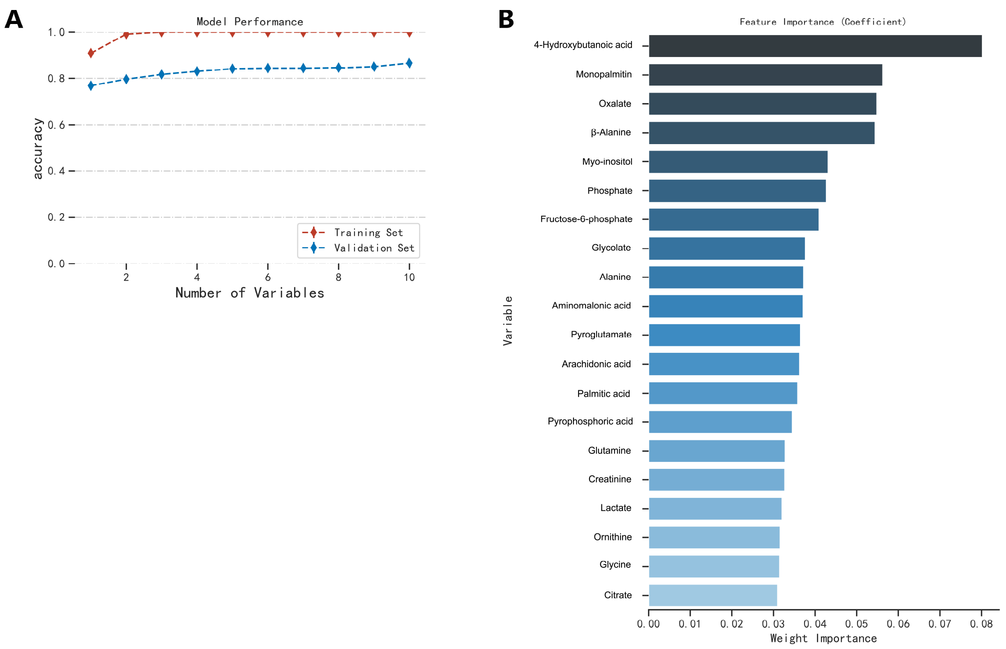

Supplementary Figure 6. Screening of predictors for the prediction model of LUAD/BLN. (A) The plot of model prediction accuracy versus the number of variables. The prediction accuracy of the developed prediction model is best when the number of predictors is 10. (B) The ranking of the importance of each variable in the model. The top ten variables were: 4-hydroxybutanoic acid, monopalmitin, oxalate, β-alanine, myo-inositol, phosphate, fructose-6-phosphate, glycolate, alanine, and aminomalonic acid.

Supplementary tables

Supplementary Table 1. Basic information of all subjects.

	LUAD	BLN	HC
Num	670	135	278
Male	267	67	163
Female	403	68	115
Age (years)	61	55	59
TNM classification			
Stage 0 (Tis)	32		
Stage IA	342		
Stage IB	38		
Stage IIA	31		
Stage IIB	32		
Stage IIIA	25		
Stage IIIB	5		
Stage IVA	7		
Stage IVB	2		
Lymph node metastasis			
Yes	73		
No	411		
Distant Metastasis			
Yes	8		
No	477		

Supplementary Table 2. The identified endogenous compounds in plasma using GC/MS system.

Identification	Derivative Type	Retention time (min)	Quant mass(m/z)	Similarity
Pyruvate	1 MEOX; 1 TMS	4.63	174	94
Lactate	2TMS	4.77	191	74
Glycolate	2TMS	4.96	177	77
Alanine	2TMS	5.27	116	98
Oxalate	2TMS	5.67	220	80
3-Hydroxybutyric acid	2TMS	5.87	191	93
Monomethylphosphate	2TMS	6.03	241	88
Valine	2TMS	6.4	218	97
4-Hydroxybutanoic acid	2TMS	6.58	233	79
Urea	2TMS	6.63	205	89
Phosphate	3TMS	6.88	314	94
Leucine	2TMS	6.9	158	84
Isoleucine	2TMS	7.07	158	95
Proline	2TMS	7.12	142	96
Glycine	3TMS	7.18	174	96
Succinate	2TMS	7.23	247	85
Glyceric acid	3TMS	7.34	292	93
Fumarate	2TMS	7.51	245	75
Serine	3TMS	7.57	204	97
Nonanoic acid	TMS	7.61	215	81
Threonine	3TMS	7.77	218	98
β-Alanine	3TMS	8.09	248	78
Aminomalonic acid	2TMS	8.36	218	80
Malate	3TMS	8.46	233	93
Aspartate	3TMS	8.68	232	96
Methionine	2TMS	8.7	176	83
Pyroglutamate	2TMS	8.72	156	95
Cysteine	3TMS	8.91	220	87
Creatinine	3TMS	8.94	115	84
Glutamate	3TMS	9.3	246	95
Phenylalanine	2TMS	9.39	218	95
Pyrophosphoric acid	4TMS	9.52	451	84
Asparagine	3TMS	9.6	231	94
Glutamine	3TMS	10.19	156	93
Citrate	4TMS	10.42	273	86
Ornithine	4TMS	10.44	142	97
Fructose	5TMS	10.76	307	89
Glucose	5TMS	10.82	319	83

Lysine	4TMS	10.98	317	93
Tyrosine	3TMS	11.08	218	96
Palmitelaidic acid	TMS	11.52	311	86
Palmitic acid	TMS	11.62	313	97
Uric acid	4TMS	11.83	441	79
Myo-Inositol	6TMS	11.83	318	80
Linoleic acid	TMS	12.39	337	97
Oleic acid	TMS	12.4	202	79
Cystine	4TMS	12.73	218	80
Fructose-6-Phosphate	1 MEOX; 6 TMS	12.95	315	79
Arachidonic acid	TMS	13.09	117	92
Monopalmitin	2TMS	13.92	371	79
Alpha-Tocopherol	TMS	16.92	502	81
Cholesterol	TMS	17.12	458	95
$^{13}C_2$-Myristic acid (IS)		10.62	287	
Myristic acid (ES)		9.96	199	
Unknown 1		4.81	190	
Unknown 2		5.09	217	
Unknown 3		5.31	204	
Unknown 4		5.99	130	
Unknown 5		6.29	228	
Unknown 6		6.48	281	
Unknown 7		6.7	157	
Unknown 8		6.74	228	
Unknown 9		6.8	184	
Unknown 10		6.98	181	
Unknown 11		7.15	256	
Unknown 12		7.23	199	
Unknown 13		7.39	285	
Unknown 14		7.67	288	
Unknown 15		7.86	174	
Unknown 16		8.04	228	
Unknown 17		8.24	200	
Unknown 18		8.26	174	
Unknown 19		8.33	200	
Unknown 20		8.52	262	
Unknown 21		8.59	180	
Unknown 22		8.81	292	
Unknown 23		8.88	260	
Unknown 24		9.09	186	
Unknown 25		9.19	211	
Unknown 26		9.46	211	
Unknown 27		9.56	273	
Unknown 28		9.63	320	

Unknown 29	9.8	347
Unknown 30	9.83	218
Unknown 31	9.87	217
Unknown 32	9.94	227
Unknown 33	10.02	193
Unknown 34	10.07	182
Unknown 35	10.34	204
Unknown 36	10.52	217
Unknown 37	10.69	217
Unknown 38	10.77	319
Unknown 39	10.81	435
Unknown 40	10.89	204
Unknown 41	11.21	305
Unknown 42	11.71	361
Unknown 43	12.1	243
Unknown 44	12.35	299
Unknown 45	12.41	339
Unknown 46	12.97	290
Unknown 47	13.21	463
Unknown 48	14.94	394
Unknown 49	15.96	227

Supplementary Table 3. Results of ROC curve analysis of differential metabolites in BLN and HC groups.

Metabolites	AUC	95%CI	P
Monopalmitin	0.86	0.81-0.90	<0.001
Succinate	0.84	0.79-0.89	<0.001
Glutamine	0.83	0.78-0.88	<0.001
Alpha-Tocopherol	0.82	0.77-0.87	<0.001
Malate	0.81	0.74-0.87	<0.001
Asparagine	0.80	0.75-0.85	<0.001
Pyroglutamate	0.80	0.73-0.86	<0.001
Citrate	0.77	0.71-0.82	<0.001
Oxalate	0.75	0.68-0.81	<0.001
Pyrophosphoric acid	0.74	0.68-0.80	<0.001
Aspartate	0.73	0.67-0.79	<0.001
Linoleic acid	0.73	0.67-0.79	<0.001
Serine	0.73	0.67-0.79	<0.001
Creatinine	0.72	0.66-0.78	<0.001
Ornithine	0.71	0.65-0.77	<0.001
Palmitic acid	0.70	0.63-0.76	<0.001
Valine	0.68	0.62-0.74	<0.001
Methionine	0.68	0.62-0.74	<0.001
Alanine	0.67	0.61-0.73	<0.001
Lactate	0.67	0.60-0.75	<0.001
Myo-Inositol	0.67	0.61-0.73	<0.001
Threonine	0.66	0.60-0.72	<0.001
Aminomalonic acid	0.65	0.59-0.71	<0.001
Tyrosine	0.65	0.59-0.71	<0.001
Leucine	0.65	0.58-0.71	<0.001
Phenylalanine	0.64	0.58-0.70	<0.001
3-Hydroxybutyric acid	0.64	0.57-0.70	<0.001
Fumarate	0.63	0.57-0.70	0.007
Proline	0.63	0.57-0.69	<0.001
Pyruvate	0.63	0.55-0.70	<0.001
4-Hydroxybutanoic acid	0.62	0.55-0.69	0.001
Isoleucine	0.61	0.55-0.67	<0.001
Arachidonic acid	0.61	0.54-0.68	0.016
Palmitelaidic acid	0.61	0.54-0.68	0.045
Uric acid	0.61	0.54-0.68	<0.001
Lysine	0.60	0.53-0.66	<0.001
Glutamate	0.59	0.52-0.65	0.014
Cysteine	0.57	0.51-0.64	0.036
β-Alanine	0.57	0.51-0.64	0.021
Glyceric acid	0.57	0.50-0.64	0.005
Glycolate	0.57	0.50-0.64	0.009
Cholesterol	0.57	0.50-0.63	0.013
Cystine	0.56	0.49-0.63	0.028

Supplementary Table 4. Results of ROC curve analysis of differential metabolites in LUAD and HC groups.

Metabolites	AUC	95%CI	P
Succinate	0.83	0.80-0.86	<0.001
Creatinine	0.80	0.77-0.84	<0.001
Alpha-Tocopherol	0.79	0.76-0.83	<0.001
Monopalmitin	0.77	0.74-0.81	<0.001
Malate	0.77	0.74-0.81	<0.001
Glutamine	0.77	0.73-0.80	<0.001
Asparagine	0.76	0.72-0.80	<0.001
Pyroglutamate	0.75	0.72-0.79	<0.001
Aminomalonic acid	0.71	0.67-0.76	<0.001
Methionine	0.71	0.67-0.75	<0.001
Serine	0.70	0.66-0.74	<0.001
Citrate	0.70	0.66-0.74	<0.001
Aspartate	0.70	0.66-0.73	<0.001
Tyrosine	0.69	0.65-0.73	<0.001
Fumarate	0.69	0.65-0.73	<0.001
Linoleic acid	0.69	0.65-0.73	<0.001
4-Hydroxybutanoic acid	0.69	0.65-0.72	<0.001
Valine	0.68	0.64-0.72	<0.001
Phenylalanine	0.68	0.63-0.72	<0.001
Leucine	0.66	0.61-0.70	<0.001
Palmitic acid	0.65	0.61-0.69	<0.001
Glycolate	0.65	0.61-0.69	<0.001
Uric acid	0.64	0.60-0.68	<0.001
Isoleucine	0.64	0.59-0.68	<0.001
3-Hydroxybutyric acid	0.63	0.59-0.68	<0.001
Pyrophosphoric acid	0.63	0.59-0.67	<0.001
Alanine	0.63	0.59-0.67	<0.001
Threonine	0.63	0.59-0.67	<0.001
Ornithine	0.62	0.57-0.66	0.006
Glutamate	0.61	0.57-0.66	0.009
Oxalate	0.61	0.57-0.65	<0.001
Glucose	0.61	0.57-0.65	0.040
Glyceric acid	0.60	0.57-0.64	<0.001
Lactate	0.60	0.57-0.64	0.002
Glycine	0.60	0.56-0.65	<0.001
Pyruvate	0.59	0.56-0.63	0.038
Palmitelaidic acid	0.59	0.55-0.63	0.008
Proline	0.59	0.54-0.63	0.032
Oleic acid	0.58	0.53-0.63	0.046
Fructose-6-Phosphate	0.57	0.52-0.61	<0.001
Cholesterol	0.56	0.51-0.60	0.003
Monomethylphosphate	0.53	0.49-0.57	0.017

Supplementary Table 5. Results of ROC curve analysis of differential metabolites in LUAD and BLN groups.

Metabolites	AUC	95%CI	P
Myo-Inositol	0.64	0.59-0.70	<0.001
Oxalate	0.63	0.57-0.69	<0.001
Monopalmitin	0.62	0.56-0.68	<0.001
Glycine	0.60	0.55-0.65	<0.001
Creatinine	0.60	0.54-0.66	<0.001
Ornithine	0.59	0.54-0.65	<0.001
Pyrophosphoric acid	0.59	0.53-0.64	<0.001
Citrate	0.59	0.53-0.64	0.008
4-Hydroxybutanoic acid	0.58	0.53-0.64	<0.001
Arachidonic acid	0.58	0.52-0.63	0.003
Aminomalonic acid	0.58	0.52-0.63	0.018
Fructose-6-Phosphate	0.57	0.51-0.63	0.005
Glutamine	0.57	0.51-0.63	0.003
β-Alanine	0.57	0.51-0.63	0.017
Lysine	0.56	0.50-0.62	<0.001
Lactate	0.56	0.50-0.62	<0.001
Glycolate	0.56	0.50-0.62	0.020
Palmitic acid	0.55	0.49-0.61	0.027
Phosphate	0.55	0.49-0.62	0.004
Malate	0.55	0.49-0.61	0.018
Asparagine	0.54	0.49-0.60	0.002
Alanine	0.54	0.48-0.60	0.004
Serine	0.54	0.48-0.60	0.004
Pyroglutamate	0.54	0.48-0.60	0.012
Proline	0.54	0.48-0.60	0.012
Threonine	0.53	0.48-0.59	0.025

Supplementary Table 6. Calculated ORs of metabolites associated with LUAD progression.

Metabolites	Early-stage vs. HC						Advanced-stage vs. Early-stage					
	OR	95%CI	P-value	OR (adjusted)	95%CI (adjusted)	P-value (adjusted)	OR	95%CI	P-value	OR (adjusted)	95%CI (adjusted)	P-value (adjusted)
Myo-Inositol	0.506	[0.338, 0.758]	0.001	0.427	[0.273, 0.666]	<0.001	0.375	[0.157, 0.899]	0.028	0.358	[0.146, 0.876]	0.025
Ornithine	0.646	[0.527, 0.790]	<0.001	0.726	[0.588, 0.896]	0.003	0.617	[0.418, 0.909]	0.015	0.567	[0.379, 0.847]	0.006
Glutamine	0.064	[0.037, 0.111]	<0.001	0.077	[0.043, 0.137]	<0.001	0.146	[0.050, 0.424]	<0.001	0.112	[0.037, 0.337]	<0.001
Asparagine	0.295	[0.220, 0.396]	<0.001	0.335	[0.246, 0.455]	<0.001	0.52	[0.272, 0.994]	0.048	0.461	[0.237, 0.896]	0.022
Pyrophosphoric acid	0.351	[0.239, 0.515]	<0.001	0.401	[0.271, 0.593]	<0.001	0.526	[0.336, 0.822]	0.005	0.513	[0.322, 0.817]	0.005
Threonine	0.534	[0.362, 0.788]	0.002	0.699	[0.471, 1.037]	0.075	0.382	[0.150, 0.974]	0.044	0.304	[0.114, 0.811]	0.017
Oxalate	2.042	[1.412, 2.951]	<0.001	1.721	[1.184, 2.501]	0.004	4.466	[1.973, 10.110]	<0.001	5.107	[2.213, 11.783]	<0.001

All ORs were adjusted for age and gender.

Supplementary Table 7. Differential metabolites and their ORs were calculated in lung adenocarcinoma patients with lymphatic metastases versus those with non-lymphoid metastases.

Metabolites	N0 (n=411)	N1+2+3 (n=73)	P-value (t-test)	OR	95%CI	P-value	OR (adjusted)	95%CI (adjusted)	P-value (adjusted)
Oxalate	5.44±0.22	5.54±0.15	<0.001	3.746	[2.023, 6.936]	<0.001	3.741	[2.001, 6.994]	<0.001
Glyceric acid	3.99±0.16	4.04±0.16	0.027	1.623	[1.057, 2.492]	0.027	1.588	[1.029, 2.451]	0.037
Nonanoic acid	3.67±0.24	3.75±0.29	0.028	1.356	[1.065, 1.728]	0.014	1.348	[1.057, 1.720]	0.016
Arachidonic acid	4.24±0.22	4.32±0.25	0.005	1.58	[1.136, 2.197]	0.007	1.588	[1.131, 2.230]	0.008
Serine	5.46±0.13	5.42±0.13	0.005	0.325	[0.149, 0.709]	0.005	0.334	[0.153, 0.730]	0.006
Methionine	4.65±0.11	4.62±0.12	0.03	0.465	[0.234, 0.924]	0.029	0.456	[0.223, 0.933]	0.031
Pyrophosphoric acid	5.04±0.19	4.92±0.21	<0.001	0.454	[0.300, 0.686]	<0.001	0.464	[0.306, 0.703]	<0.001
Asparagine	4.31±0.16	4.24±0.17	0.002	0.453	[0.272, 0.752]	0.002	0.458	[0.273, 0.766]	0.003
Glutamine	5.94±0.13	5.87±0.14	<0.001	0.19	[0.084, 0.430]	<0.001	0.188	[0.082, 0.432]	<0.001
Ornithine	4.92±0.28	4.84±0.28	0.016	0.69	[0.510, 0.934]	0.016	0.68	[0.500, 0.926]	0.014
Uric acid	5.71±0.18	5.67±0.19	0.044	0.576	[0.337, 0.985]	0.044	0.552	[0.307, 0.991]	0.047
Myo-Inositol	4.71±0.12	4.67±0.16	0.005	0.37	[0.189, 0.723]	0.004	0.346	[0.174, 0.689]	0.003
Phosphate	6.12±0.14	6.07±0.12	0.007	0.243	[0.087, 0.676]	0.007	0.228	[0.079, 0.654]	0.006
Proline	5.59±0.33	5.44±0.50	0.019	0.756	[0.626, 0.914]	0.004	0.758	[0.627, 0.917]	0.004
Glycine	4.98±0.20	4.89±0.23	<0.001	0.48	[0.314, 0.734]	0.001	0.484	[0.316, 0.739]	0.001
Creatinine	4.66±0.16	4.59±0.20	0.006	0.464	[0.295, 0.729]	0.001	0.47	[0.299, 0.740]	0.001
Lysine	4.51±0.18	4.46±0.19	0.015	0.597	[0.393, 0.906]	0.015	0.598	[0.393, 0.912]	0.017

The data were log10 transformed and expressed as mean ± SD. All ORs were adjusted for age and gender.

Supplementary Table 8. Differential metabolites and their ORs were calculated in patients with lung adenocarcinoma with distant metastases versus those with non-distant metastases.

Metabolites	M0 (n=489)	M1 (n=8)	P-value (t-test)	OR	95%CI	P-value	OR (adjusted)	95%CI (adjusted)	P-value (adjusted)
Oxalate	5.45±0.21	5.63±0.13	0.005	16.961	[2.255, 127.573]	0.006	19.935	[2.637, 150.727]	0.004
Arachidonic acid	4.25±0.22	4.48±0.15	0.003	3.828	[1.484, 9.874]	0.005	4.736	[1.712, 13.105]	0.003
Glycine	4.97±0.20	4.82±0.19	0.036	0.288	[0.087, 0.947]	0.04	0.276	[0.081, 0.938]	0.039
Glycolate	3.92±0.15	3.78±0.13	0.023	0.326	[0.129, 0.819]	0.017	0.307	[0.122, 0.773]	0.012

The data were log10 transformed and expressed as mean ± SD. All ORs were adjusted for age and ge

Supplementary Table 9. Performance of various machine learning models in lung nodules and healthy controls.

Cohorts	Models	Accuracy (95%CI)	AUC (95%CI)	Cutoff (95%CI)	Sensitivity(95%CI)	Specificity (95%CI)	PPV (95%CI)	NPV (95%CI)	F1-score (95%CI)	Kappa (95%CI)
Training	Random Forest	1.00 (1.00-1.00)	1.00 (NaN-NaN)	0.60 (0.58-0.63)	1.00 (1.00-1.00)	1.00 (1.00-1.00)	1.00 (1.00-1.00)	1.00 (1.00-1.00)	1.00 (1.00-1.00)	1.00 (1.00-1.00)
	XGBoost	0.90 (0.89-0.90)	0.97 (0.96-0.98)	0.76 (0.75-0.78)	0.88 (0.87-0.89)	0.95 (0.94-0.96)	0.98 (0.98-0.99)	0.72 (0.71-0.74)	0.93 (0.92-0.93)	0.75 (0.74-0.76)
	AdaBoost	0.86 (0.85-0.87)	0.95 (0.93-0.96)	0.52 (0.52-0.52)	0.84 (0.82-0.86)	0.91 (0.89-0.93)	0.96 (0.96-0.97)	0.67 (0.64-0.69)	0.90 (0.89-0.91)	0.67 (0.65-0.68)
	MLP	0.63 (0.58-0.68)	0.72 (0.68-0.75)	0.66 (0.63-0.70)	0.59 (0.51-0.68)	0.76 (0.70-0.82)	0.88 (0.87-0.90)	0.40 (0.36-0.44)	0.70 (0.64-0.76)	0.27 (0.22-0.33)
	SVM	0.76 (0.74-0.77)	0.82 (0.79-0.85)	0.99 (0.99-1.00)	0.76 (0.73-0.79)	0.76 (0.73-0.79)	0.90 (0.89-0.91)	0.52 (0.50-0.55)	0.82 (0.81-0.84)	0.45 (0.43-0.47)
	KNN	0.26 (0.25-0.26)	1.00 (NaN-NaN)	1.00 (1.00-1.00)	1.00 (1.00-1.00)	1.00 (1.00-1.00)	NaN (NaN-NaN)	0.26 (0.25-0.26)	NaN (NaN-NaN)	0.00 (0.00-0.00)
	GNB	0.86 (0.85-0.86)	0.92 (0.90-0.94)	0.73 (0.71-0.75)	0.87 (0.86-0.87)	0.83 (0.82-0.84)	0.94 (0.93-0.94)	0.68 (0.66-0.69)	0.90 (0.90-0.90)	0.65 (0.64-0.65)
Validation	Random Forest	0.85 (0.83-0.86)	0.89 (0.84-0.94)	0.60 (0.58-0.63)	0.83 (0.79-0.87)	0.81 (0.77-0.85)	0.90 (0.89-0.91)	0.68 (0.64-0.72)	0.87 (0.84-0.89)	0.59 (0.54-0.63)
	XGBoost	0.82 (0.80-0.83)	0.88 (0.83-0.93)	0.76 (0.75-0.78)	0.79 (0.75-0.83)	0.84 (0.80-0.88)	0.91 (0.89-0.92)	0.63 (0.59-0.66)	0.84 (0.82-0.87)	0.56 (0.52-0.59)
	AdaBoost	0.81 (0.79-0.83)	0.89 (0.84-0.94)	0.52 (0.52-0.52)	0.85 (0.81-0.88)	0.81 (0.76-0.86)	0.93 (0.91-0.94)	0.58 (0.55-0.61)	0.88 (0.86-0.90)	0.54 (0.50-0.58)
	MLP	0.63 (0.57-0.68)	0.70 (0.62-0.77)	0.66 (0.63-0.70)	0.58 (0.51-0.66)	0.79 (0.73-0.86)	0.88 (0.84-0.91)	0.41 (0.36-0.46)	0.69 (0.63-0.75)	0.27 (0.21-0.33)
	SVM	0.75 (0.73-0.78)	0.83 (0.77-0.89)	0.99 (0.99-1.00)	0.73 (0.67-0.79)	0.82 (0.77-0.87)	0.91 (0.89-0.92)	0.51 (0.46-0.55)	0.81 (0.77-0.85)	0.44 (0.40-0.49)
	KNN	0.26 (0.24-0.27)	0.89 (0.83-0.94)	1.00 (1.00-1.00)	0.82 (0.81-0.84)	0.85 (0.81-0.88)	NaN (NaN-NaN)	0.26 (0.24-0.27)	NaN (NaN-NaN)	0.00 (0.00-0.00)
	GNB	0.84 (0.82-0.85)	0.91 (0.87-0.95)	0.73 (0.71-0.75)	0.86 (0.82-0.90)	0.83 (0.79-0.88)	0.92 (0.91-0.93)	0.66 (0.62-0.71)	0.89 (0.87-0.91)	0.61 (0.57-0.64)

Abbreviations: PPV, positive predictive value; NPV, negative predictive value.

Supplementary Table 10. Performance of various machine learning models in LUAD and BLN groups.

Cohorts	Models	Accuracy (95%CI)	AUC (95%CI)	Cutoff (95%CI)	Sensitivity (95%CI)	Specificity (95%CI)	PPV (95%CI)	NPV (95%CI)	F1-score (95%CI)	Kappa (95%CI)
Training	Random Forest	1.00 (1.00-1.00)	1.00 (NaN-NaN)	0.75 (0.73-0.77)	1.00 (1.00-1.00)	1.00 (1.00-1.00)	1.00 (1.00-1.00)	0.99 (0.98-0.99)	1.00 (1.00-1.00)	0.99 (0.99-0.99)
	XGBoost	0.85 (0.83-0.86)	0.94 (0.92-0.96)	0.72 (0.71-0.74)	0.84 (0.82-0.86)	0.90 (0.88-0.92)	0.98 (0.97-0.98)	0.53 (0.50-0.56)	0.90 (0.89-0.91)	0.58 (0.55-0.61)
	AdaBoost	0.73 (0.70-0.75)	0.86 (0.83-0.90)	0.58 (0.58-0.59)	0.70 (0.66-0.73)	0.90 (0.87-0.93)	0.97 (0.96-0.98)	0.37 (0.35-0.39)	0.81 (0.79-0.83)	0.38 (0.35-0.40)
	GNB	0.63 (0.58-0.68)	0.70 (0.65-0.76)	0.63 (0.46-0.80)	0.61 (0.53-0.69)	0.71 (0.64-0.79)	0.92 (0.91-0.93)	0.28 (0.25-0.30)	0.73 (0.68-0.78)	0.20 (0.17-0.23)
	MLP	0.50 (0.45-0.55)	0.59 (0.53-0.65)	0.71 (0.67-0.76)	0.46 (0.39-0.54)	0.71 (0.65-0.78)	0.89 (0.88-0.91)	0.21 (0.20-0.22)	0.60 (0.53-0.67)	0.09 (0.07-0.11)
	SVM	0.59 (0.55-0.64)	0.69 (0.63-0.74)	0.78 (0.52-1.03)	0.56 (0.50-0.63)	0.74 (0.67-0.82)	0.92 (0.90-0.93)	0.26 (0.25-0.28)	0.69 (0.64-0.74)	0.18 (0.17-0.19)
	KNN	0.17 (0.16-0.17)	1.00 (NaN-NaN)	1.00 (1.00-1.00)	1.00 (1.00-1.00)	1.00 (1.00-1.00)	NaN (NaN-NaN)	0.17 (0.16-0.17)	NaN (NaN-NaN)	0.00 (0.00-0.00)
Validation	Random Forest	0.73 (0.70-0.77)	0.76 (0.69-0.84)	0.75 (0.73-0.77)	0.71 (0.66-0.75)	0.73 (0.69-0.76)	0.91 (0.89-0.92)	0.34 (0.30-0.37)	0.79 (0.76-0.83)	0.26 (0.23-0.30)
	XGBoost	0.73 (0.70-0.76)	0.75 (0.68-0.83)	0.72 (0.71-0.74)	0.63 (0.57-0.68)	0.80 (0.72-0.87)	0.90 (0.89-0.91)	0.34 (0.31-0.37)	0.74 (0.70-0.78)	0.27 (0.24-0.30)
	AdaBoost	0.65 (0.63-0.68)	0.72 (0.64-0.80)	0.58 (0.58-0.59)	0.64 (0.58-0.71)	0.76 (0.68-0.83)	0.91 (0.89-0.92)	0.28 (0.25-0.30)	0.75 (0.70-0.79)	0.20 (0.17-0.23)
	GNB	0.60 (0.55-0.64)	0.67 (0.59-0.76)	0.63 (0.46-0.80)	0.61 (0.52-0.69)	0.70 (0.64-0.76)	0.88 (0.86-0.91)	0.25 (0.23-0.27)	0.71 (0.65-0.77)	0.13 (0.12-0.15)
	MLP	0.50 (0.44-0.55)	0.60 (0.51-0.69)	0.71 (0.67-0.76)	0.48 (0.34-0.62)	0.75 (0.63-0.86)	0.88 (0.86-0.89)	0.21 (0.19-0.23)	0.59 (0.46-0.72)	0.08 (0.05-0.11)
	SVM	0.57 (0.52-0.62)	0.65 (0.57-0.74)	0.78 (0.52-1.03)	0.55 (0.42-0.69)	0.71 (0.57-0.86)	0.90 (0.88-0.92)	0.21 (0.19-0.24)	0.66 (0.55-0.77)	0.11 (0.08-0.14)
	KNN	0.17 (0.15-0.18)	0.70 (0.61-0.79)	1.00 (1.00-1.00)	0.63 (0.58-0.69)	0.74 (0.68-0.80)	NaN (NaN-NaN)	0.17 (0.15-0.18)	NaN (NaN-NaN)	0.00 (0.00-0.00)

Abbreviations: PPV, positive predictive value; NPV, negative predictive value.
